# The replicative lifespan‐extending deletion of *SGF73* results in altered ribosomal gene expression in yeast

**DOI:** 10.1111/acel.12611

**Published:** 2017-05-31

**Authors:** Amanda G. Mason, Renee M. Garza, Mark A. McCormick, Bhumil Patel, Brian K. Kennedy, Lorraine Pillus, Albert R. La Spada

**Affiliations:** ^1^ Department of Pediatrics University of California, San Diego La Jolla CA USA; ^2^ Division of Biological Sciences University of California, San Diego La Jolla CA USA; ^3^ Moores Cancer Center University of California, San Diego La Jolla CA USA; ^4^ Buck Institute for Research on Aging Novato CA USA; ^5^ Department of Biochemistry University of Washington Seattle WA USA; ^6^ Departments of Cellular & Molecular Medicine and Neurosciences University of California, San Diego La Jolla CA USA; ^7^ Institute for Genomic Medicine University of California, San Diego La Jolla CA USA; ^8^ Sanford Consortium for Regenerative Medicine University of California, San Diego La Jolla CA USA; ^9^ Rady Children's Hospital San Diego CA USA; ^10^ Present address: Department of Human Genetics Leiden University Medical Center Leiden The Netherlands

**Keywords:** genome‐wide occupancy, longevity gene, Neurodegeneration, replicative lifespan, Sgf73, yeast

## Abstract

Sgf73, a core component of SAGA, is the yeast orthologue of ataxin‐7, which undergoes CAG–polyglutamine repeat expansion leading to the human neurodegenerative disease spinocerebellar ataxia type 7 (SCA7). Deletion of *SGF73* dramatically extends replicative lifespan (RLS) in yeast. To further define the basis for Sgf73‐mediated RLS extension, we performed ChIP‐Seq, identified 388 unique genomic regions occupied by Sgf73, and noted enrichment in promoters of ribosomal protein (RP)‐encoding genes. Of 388 Sgf73 binding sites, 33 correspond to 5′ regions of genes implicated in RLS extension, including 20 genes encoding RPs. Furthermore, half of Sgf73‐occupied, RLS‐linked RP genes displayed significantly reduced expression in *sgf73Δ* mutants, and double null strains lacking *SGF73* and a Sgf73‐regulated, RLS‐linked RP gene exhibited no further increase in replicative lifespan. We also found that *sgf73Δ* mutants display altered acetylation of Ifh1, an important regulator of RP gene transcription. These findings implicate altered ribosomal protein expression in *sgf73Δ* yeast RLS and highlight altered acetylation as a pathway of relevance for SCA7 neurodegeneration.

## Introduction

The SAGA (Spt‐Ada‐Gcn5 Acetyltransferase) complex is a major transcriptional coactivator complex responsible for regulation of a large number of yeast genes, the majority of which are stress‐induced (Huisinga & Pugh, 2004). Additionally, SAGA regulates many actively transcribed yeast genes, and the cognate orthologous STAGA (Spt3‐Taf9‐Gcn5 acetyltransferase) complex regulates the expression of numerous mammalian genes (Bonnet *et al*., 2014), suggesting its broad significance for transcription regulation. The SAGA/STAGA complex is recruited to its target genes by many transcription factors, and through both its Gcn5/GCN5 acetylation and Ubp8/USP22 deubiquitination activities at promoters, it facilitates transcription activation and elongation [reviewed in (Koutelou *et al*., 2010)]. The SAGA complex has four distinct modules responsible for acetyltransferase activity, deubiquitination activity, complex recruitment, and architecture of the complex. Sgf73 is a subunit of SAGA (Helmlinger *et al*., 2004; Timmers & Tora, 2005), which acts in the Ubp8 deubiquitinase module (DUBm), linking the core SAGA Gcn5‐mediated acetylation activity to the Ubp8 DUBm (Kohler *et al*., 2008; Zhao *et al*., 2008; Lee *et al*., 2009; Morgan *et al*., 2016).

Deletion of *SGF73* results in a dramatic extension in yeast RLS (McCormick *et al*., 2014). RLS is defined as the number of daughter cells that a mother cell produces before senescence. *SGF73* is required for Ubp8‐mediated histone H2B deubiquitination, with *sgf73Δ* strains having elevated H2BK123 ubiquitination, as is seen in *ubp8Δ* strains (Kohler *et al*., 2008). This increase in ubiquitinated H2BK123 contributes to the RLS extension of *sgf73Δ* mutants, as strains that lack *UBP8* are also long‐lived, and strains harboring the H2B‐K123R mutation, which cannot be ubiquitinated, are short‐lived (McCormick *et al*., 2014).

The RLS extension in *sgf73Δ* mutants is Sir2 dependent, and *sgf73Δ* strains have altered Sir2‐related activities (McCormick *et al*., 2014). *SIR2* encodes a class III NAD+‐dependent deacetylase that has been implicated in both the regulation of lifespan and the benefits of caloric restriction in a variety of species, making it a central focus of aging research. Overexpression of *SIRT1*, the closest mammalian orthologue of *SIR2,* has varying effects on senescence depending on the cell type, further underscoring the important role of the sirtuins in aging (Langley *et al*., 2002; Chua *et al*., 2005). Additionally, yeast Sir2 is required for telomeric and ribosomal DNA (rDNA) silencing, and suppression of homologous recombination within the rDNA repeats [reviewed in (Kueng *et al*., 2013)]. Inappropriate rDNA recombination results in the accumulation of rDNA circles, which is one factor correlated with aging in yeast (reviewed in (Blander & Guarente, 2004). SAGA has been shown to play a role in the asymmetric retention of rDNA circles in the mother cell by tethering the rDNA circles to nuclear pore complexes. *SGF73* deletion reduces rDNA circle retention (Denoth‐Lippuner *et al*., 2014).

Ribosomal biogenesis is another key determinant of aging. In particular, a reduction in 60S ribosomal subunits promotes lifespan extension in yeast (Steffen *et al*., 2008), and a reduction in both the 40S and 60S ribosomal subunits in *Caenorhabditis elegans* yields longer lifespan in this metazoan model organism (Chen *et al*., 2007; Curran & Ruvkun, 2007; Hansen *et al*., 2007). Multiple signaling pathways regulate ribosomal biogenesis in response to nutrients and stress. One of these is the nutrient‐sensing target of rapamycin (TOR) signaling pathway that regulates the interacting coactivator Ifh1 (Interacts with Forkhead) and corepressor Crf1 (Co‐Repressor with FHL1) of the Forkhead‐like transcription factor (Fhl1) (Martin *et al*., 2004). The dynamic process of ribosomal protein gene transcription is regulated by the interaction of Ifh1 with Fhl1, which is bound at RP gene promoters (Warner, 1999). Ifh1 is first recruited to ribosomal gene promoters upon nutrient stimulus, with acetylation being a key regulator of Ifh1 activity (Schawalder *et al*., 2004). Ifh1 activity is decreased upon Gcn5‐mediated acetylation, whereas Sir2 and another sirtuin, Hst1, deacetylate Ifh1, resulting in increased activity. It is this on–off cycle that contributes to proper regulation of RP gene transcription (Downey *et al*., 2013).

The human counterpart of Sgf73 is ataxin‐7, and the gene encoding this protein causes the autosomal dominant neurodegenerative disorder spinocerebellar ataxia type 7 (SCA7), upon CAG–polyglutamine repeat expansion. Ataxin‐7 is a member of the mammalian transcription coactivator complex STAGA, where it plays a similar role to its yeast DUBm counterpart (Martinez *et al*., 2001; Helmlinger *et al*., 2004). Ataxin‐7 has an amino‐terminal CAG/polyglutamine tract that normally ranges from four to 35 repeats. However, when this tract expands to repeat lengths greater than 37, humans develop SCA7 (David *et al*., 1997; Stevanin *et al*., 2000). SCA7 is thus one of the nine heritable CAG–polyglutamine repeat expansion disorders that all result in expanded polyglutamine tracts in different proteins. Accumulation of polyglutamine‐expanded ataxin‐7 protein leads to the dysfunction and death of neurons in the retina, cerebellum, and brainstem, causing blindness, progressive loss of coordination, and premature death (Lebre & Brice, 2003).

To probe the molecular mechanisms of Sgf73/ataxin‐7 function, we used a ChIP‐Seq approach, identifying 388 genomic regions bound by Sgf73. Notably, we found an enrichment of binding in the 5′ promoter regions of genes encoding ribosomal proteins (RPs). Of the Sgf73‐occupied yeast genomic regions, 33 corresponded to the 5′ regions of genes previously linked to RLS extension, with the majority of these genes encoding RPs. We found that deletion of *SGF73* altered the expression of Sgf73‐occupied RP genes at baseline and upon rapamycin stress. Furthermore, we found that double null strains of yeast lacking SGF73 and a Sgf73‐regulated RLS‐linked RP gene did not exhibit any further increase in RLS. Finally, we noted altered acetylation of the TOR target Ifh1 in *sgf73Δ* mutants. Our results reveal Sgf73 target genes that are integral to pathways of aging regulation in yeast and may highlight pathways regulating mammalian aging and SCA7 neurodegeneration in humans.

## Results

### Sgf73 ChIP‐Seq analysis yields 388 unique genomic occupancy sites

**Figure 1:**
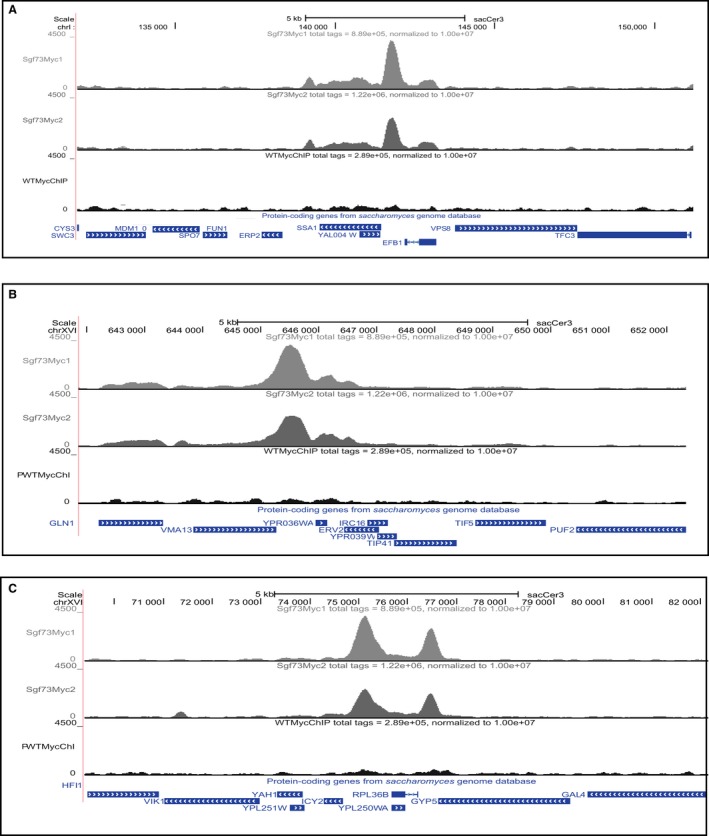
Representative genome tracks of Sgf73‐bound peaks. Visual inspection of genome tracks of reads from significant peaks shows correct assignment of called peaks, uniformity between Sgf73 ChIP replicates, and significance over background. The three top‐ranked peaks (highest peak scores) are presented: (A) SSA1, (B) YPR036W‐A, and (C) ICY2. Note that in the ICY2 track image (C), the significant peak for RPL36B occurs 3′ to ICY2.

To perform ChIP on Sgf73, we integrated a carboxy‐terminal 13‐Myc epitope. Integration was verified by molecular genetic analysis, while tagged protein production and ChIP‐tagged protein recovery were confirmed by immunoblotting (Fig. S1). In addition, functionality of the tagged protein was confirmed using standard growth assays that demonstrated that the 13‐Myc tag did not interfere with Sgf73 function (Fig. S2). Sgf73 ChIP'd DNA was processed into libraries, and single‐end sequencing was performed to generate ~17 million 50‐bp reads per sample. Raw sequence reads were aligned to the *Saccharomyces cerevisiae* S288C genome 3 (sacCer3). Significant peaks were identified using HOMER (Hypergeometric Optimization of Motif EnRichment) v4.2 (Heinz *et al*., 2010). These were defined as a collection of sequence reads, collectively mapping to genomic locations at a significantly higher density than background and surrounding genomic areas (Fig. 1). Our Sgf73 ChIP‐Seq analysis identified 389 significant peaks (Table S1), with two significant peaks positioned 5′ to the same transcriptional start site (TSS), resulting in 388 unique occupancy sites. The tag counts, which correspond to the number of unique reads mapping to the indicated genomic region, were used to rank the peaks.

### Genes encoding ribosomal proteins (RPs) are enriched for Sgf73 occupancy

**Figure 2:**
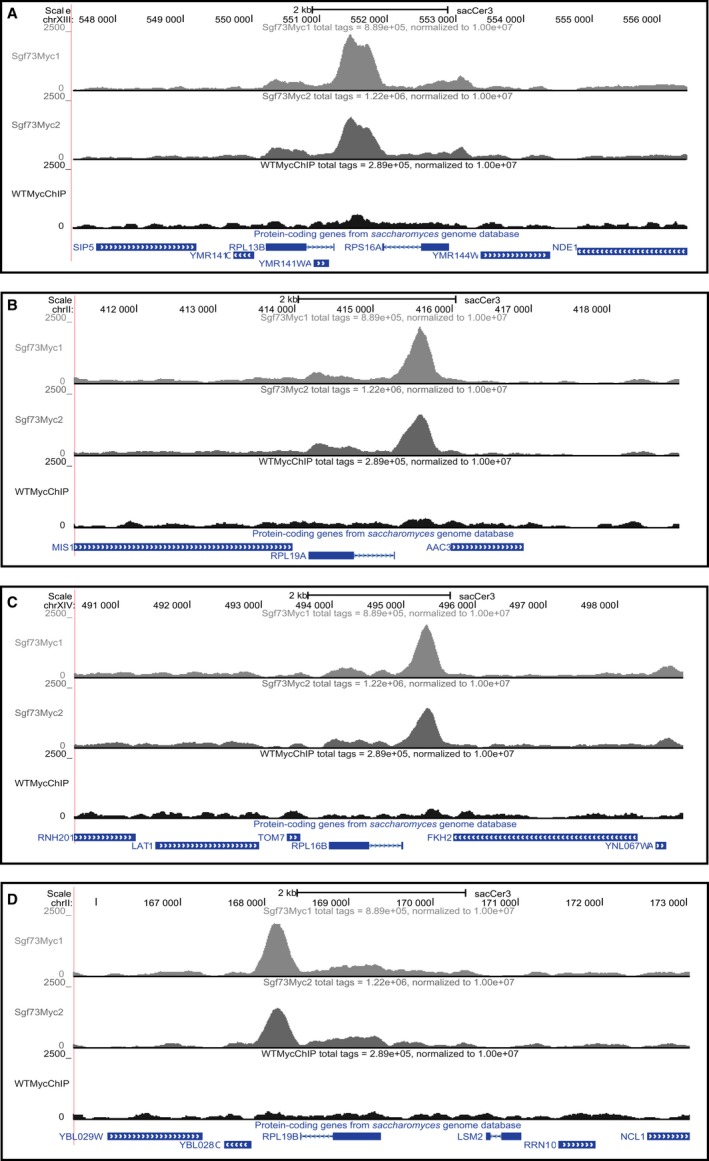
Representative genome tracks of RLS‐linked RP Sgf73 occupancy peaks. Many Sgf73‐occupied peaks that are linked to genes involved in RLS extension upon deletion map 5′ to RP genes. Shown are genome tracks of representative peaks of the following RLS‐linked RP genes: (A) RPL13B, (B) RPL19A, (C) RPL16B, and (D) RPL19B.

To understand the most significant genes and pathways affected by Sgf73, we performed enrichment analyses using WebGestalt (Zhang *et al*., 2005; Wang *et al*., 2013), followed by Gene Ontology (GO) and the Kyoto Encyclopedia of Genes and Genomes (KEGG) (Kanehisa & Goto, 2000; Kanehisa *et al*., 2012, 2014) analysis to reveal gene families and pathways that were enriched in the set of Sgf73‐occupied genes (Fig. S3A). Most striking from this analysis is the enrichment of ‘ribosome’ in GO terms and, significantly, ‘ribosome’ is the only enriched KEGG pathway (*P* = 1.96e‐34), with 57 of the 388 peaks (~14.5%) displaying Sgf73 occupancy proximal to a RP gene (Table 1). Survey of the ChIP‐Seq tracks confirmed the presence of Sgf73 RP occupancy peaks (Fig. 2). Of the 57 ribosomal protein genes, 36 encode large ribosomal subunits, 20 encode small ribosomal subunits, and one encodes a ribosomal stalk protein. It is noteworthy that numerous studies have linked ribosomal biogenesis to aging and have established that reduced expression of large ribosomal subunits can yield dramatically increased RLS (Kaeberlein *et al*., 2005; Steffen *et al*., 2008). Our findings thus indicate that Sgf73 loss of function may promote increased RLS by altering ribosome regulation.

**Table 1:** Ribosomal protein genes with 5′ occupancy by Sgf73

Gene symbol	Gene name	Ensembl gene stable ID	Entrez ID
RPL1B	60S ribosomal protein L1	YGL135W	852742
RPL2A	60S ribosomal protein L2	YFR031C‐A	850590
RPL5	60S ribosomal protein L5	YPL131W	855972
RPL6A	60S ribosomal protein L6‐A	YML073C	854902
RPL6B	60S ribosomal protein L6‐B	YLR448W	851169
RPL7A	60S ribosomal protein L7‐A	YGL076C	852804
RPL8A	60S ribosomal protein L8‐A	YHL033C	856352
RPL10	60S ribosomal protein L10	YLR075W	850764
RPL13B	60S ribosomal protein L13‐B	YMR142C	855173
RPL14A	60S ribosomal protein L14‐A	YKL006W	853864
RPL16B	60S ribosomal protein L16‐B	YNL069C	855655
RPL17A	60S ribosomal protein L17‐A	YKL180W	853674
RPL19A	60S ribosomal protein L19	YBR084C‐A	852379
RPL19B	60S ribosomal protein L19	YBL027W	852254
RPL20A	60S ribosomal protein L20‐A	YMR242C	855283
RPL20B	60S ribosomal protein L20	YOR312C	854489
RPL22A	60S ribosomal protein L22‐A	YLR061W	850750
RPL23A	60S ribosomal protein L23	YBL087C	852191
RPL23B	60S ribosomal protein L23	YER117W	856853
RPL26A	60S ribosomal protein L26‐A	YLR344W	851058
RPL27B	60S ribosomal protein L27‐B	YDR471W	852082
RPL28	60S ribosomal protein L28	YGL103W	852775
RPL30	60S ribosomal protein L30	YGL030W	852853
RPL31A	60S ribosomal protein L31‐A	YDL075W	851484
RPL33A	60S ribosomal protein L33‐A	YPL143W	855960
RPL34B	60S ribosomal protein L34‐B	YIL052C	854759
RPL35A	60S ribosomal protein L35	YDL191W	851336
RPL36B	60S ribosomal protein L36‐B	YPL249C‐A	855826
RPL37B	60S ribosomal protein L37‐B	YDR500C	852111
RPL39	60S ribosomal protein L39	YJL189W	853250
RPL40A	60S ribosomal protein L40; Ubiquitin	YIL148W	854658
RPL41A	60S ribosomal protein L41	YDL184C	851344
RPL41B	60S ribosomal protein L41	YDL133C‐A	851422
RPL42A	60S ribosomal protein L42	YNL162W	855560
RPL43A	60S ribosomal protein L43	YPR043W	856156
RPL43B	60S ribosomal protein L43	YJR094W‐A	853557
RPP2A	60S acidic ribosomal protein P2‐alpha	YOL039W	854118
RPS0A	40S ribosomal protein S0‐A	YGR214W	853128
RPS0B	40S ribosomal protein S0‐B	YLR048W	850737
RPS1A	YLR441C	YLR441C	851162
RPS3	40S ribosomal protein S3	YNL178W	855543
RPS5	40S ribosomal protein S5	YJR123W	853587
RPS7A	40S ribosomal protein S7‐A	YOR096W	854263
RPS7B	40S ribosomal protein S7‐B	YNL096C	855628
RPS8B	40S ribosomal protein S8	YER102W	856839
RPS9A	40S ribosomal protein S9‐A	YPL081W	856024
RPS9B	40S ribosomal protein S9‐B	YBR189W	852487
RPS11B	40S ribosomal protein S11	YBR048W	852337
RPS12	40S ribosomal protein S12	YOR369C	854551
RPS14A	40S ribosomal protein S14‐A	YCR031C	850397
RPS16B	40S ribosomal protein S16	YDL083C	851476
RPS17B	40S ribosomal protein S17‐B	YDR447C	852058
RPS18B	40S ribosomal protein S18	YML026C	854982
RPS19B	40S ribosomal protein S19‐B	YNL302C	855414
RPS21B	40S ribosomal protein S21‐B	YJL136C	853305
RPS24A	40S ribosomal protein S24	YER074W	856805
RPS31	40S ribosomal protein S31; Ubiquitin	YLR167W	850864

### Genes encoding large ribosomal subunits implicated in RLS extension studies are enriched for Sgf73 binding

Deletion of *SGF73* results in a dramatic extension in RLS. As a core component of the SAGA transcription coactivator complex, Sgf73 loss of function may alter the expression of genes catalogued to promote RLS extension. We considered the results of a recent study in which all nonessential yeast deletion mutants were subjected to RLS analysis (McCormick *et al*., 2015). Comparison of Sgf73 genomic occupancy sites with these 238 yeast genes, found to significantly promote RLS upon deletion, revealed 33 uniquely shared genes (Table 2; Fig. S3B). Among these 33 genes (McCormick *et al*., 2015), 20 encode RPs, of which 18 of the 20 RP genes encode large ribosomal subunits. To confirm that these promoters are SAGA DUBm‐targeted, we performed ChIP analysis of Ubp8 and observed that Ubp8 co‐occupied 14 of the 19 RLS‐linked RP regions enriched for Sgf73 binding (Table S1 and Fig. S4). However, it should be noted that Sgf73 is known to bind DNA through its zinc finger domain (Bonnet *et al*., 2010), whereas there is no evidence for Ubp8 directly binding to DNA, which is supported by our detection of only 174 Ubp8 peaks, of which 171 are also Sgf73‐occupied (Table S2; Gene Expression Omnibus accession #GSE76461). As the yeast RLS extension single‐gene deletion survey yielded a total of 26 RP genes encoding large ribosome subunits (McCormick *et al*., 2015), and 18 of these 26 large ribosome unit genes (i.e., ~69%) exhibit Sgf73 occupancy, altered regulation of large ribosomal subunit gene expression may contribute to the extreme RLS extension phenotype observed in *sgf73Δ* yeast strains.

**Table 2:** Sgf73 occupied genes implicated in replicative lifespan extension

Chr #	Start	End	Peak score	bp to TSS	Promoter ID	Gene name	Gene description
chrXIII	388 438	388 553	2899.699951	235	YMR058W	FET3	Ferro‐02‐oxidoredutase
chrXVI	679 114	679 269	2307.100098	240	YPR064W	YPR064W	
chrlV	765 359	765 481	2291.649902	−265	YDR151C	CTH1	Cth1p
chrXIII	551 398	551 502	2158.399902	−243	YMR142C	RPL13B	Ribosomal 60S subunit protein L13B
chrII	415 546	415 648	2027.050049	−336	YBR084C‐A	RPL19A	Ribosomal 60S subunit protein L19A
chrXIV	495 272	495 401	1984.149902	−335	YNL069C	RPL16B	Ribosomal 60S subunit protein L16B
chrII	168 066	168 211	1955	−285	YBL027W	RPL19B	Ribosomal 60S subunit protein L19B
chrVII	23 427	23 542	1886.400024	−451	YGL253W	HXK2	Hexokinase 2
chrVII	254 368	254 492	1819.050049	−211	YGL135W	RPL1B	Ribosomal 60S subunit protein L1B
chrIV	321 879	321 984	1733.550049	−295	YDL075W	RPL31A	Ribosomal 60S subunit protein L31A
chrXIV	559 344	559 470	1728.550049	−405	YNL037C	IDH1	Isocitrate dehydrogenase [NAD(+)]
chrXV	253 915	254 041	1710.600098	−319	YOL039W	RPP2A	Ribosomal protein P2A
chrIX	257 361	257 495	1688.699951	−365	YIL052C	RPL34B	Ribosomal 60S subunit protein L34B
chrXIII	124 367	124 483	1627.349976	−253	YML073C	RPL6A	Ribosomal 60S subunit protein L6A
chrIV	117 431	117 534	1530.599976	−182	YDL191W	RPL35A	Ribosomal 60S subunit protein L35A
chrXV	901 425	901 527	1482.649902	−282	YOR312C	RPL20B	Ribosomal 60S subunit protein L20B
chrXII	1 028 449	1 028 574	1472.050049	−343	YLR448W	RPL6B	Ribosomal 60S subunit protein L6B
chrXIII	754 426	754 526	1431.550049	179	YMR242C	RPL20A	Ribosomal 60S subunit protein L20A
chrV	153 026	153 167	1352.5	−424	YER001W	MNN1	Mnn1p
chrXII	125 194	125 303	1343.649902	−286	YLL012W	YEH1	Yeh1p
chrII	60 934	61 043	1330.300049	−249	YBL087C	RPL23A	Ribosomal 60S subunit protein L23A
chrVII	366 078	366 180	1292.449951	−133	YGL076C	RPL7A	Ribosomal 60S subunit protein L7A
chrXV	1 028 799	1 028 904	1232.300049	−226	YOR369C	RPS12	Ribosomal 40S subunit protein S12
chrX	525 859	525 976	1129.050049	−418	YJR048W	CYC1	Cyc1p
chrIV	1 450 918	1 451 035	1095.849976	−123	YDR500C	RPL37B	Ribosomal 60S subunit protein L37B
chrVII	1 050 308	1 050 489	1042.550049	−440	YGR279C	SCW4	Scw4p
chrXVI	280 095	280 222	954.099976	−322	YPL144W	POC4	Poc4p
chrXII	818 597	818 710	822.75	−659	YLR344W	RPL26A	Ribosomal 60S subunit protein L26A
chrXII	514 958	515 073	775.25	−247	YLR180W	SAM1	Methionine adenosyltransferase
chrX	607 973	608 113	763.5	−262	YJR094W‐A	RPL43B	Rpl43 bp
chrXII	263 015	263 122	756.400024	−126	YLR061W	RPL22A	Ribosomal 60S subunit protein L22A
chrII	697 587	697 702	604.550049	−342	YBR238C	YBR238C	Hypothetical protein
chrVII	976 969	977 114	599.450012	−295	YGR243W	FMP43	Fmp43p

### RP‐occupied genes Sgf73 have reduced expression in *sgf73Δ* mutants

**Figure 3:**
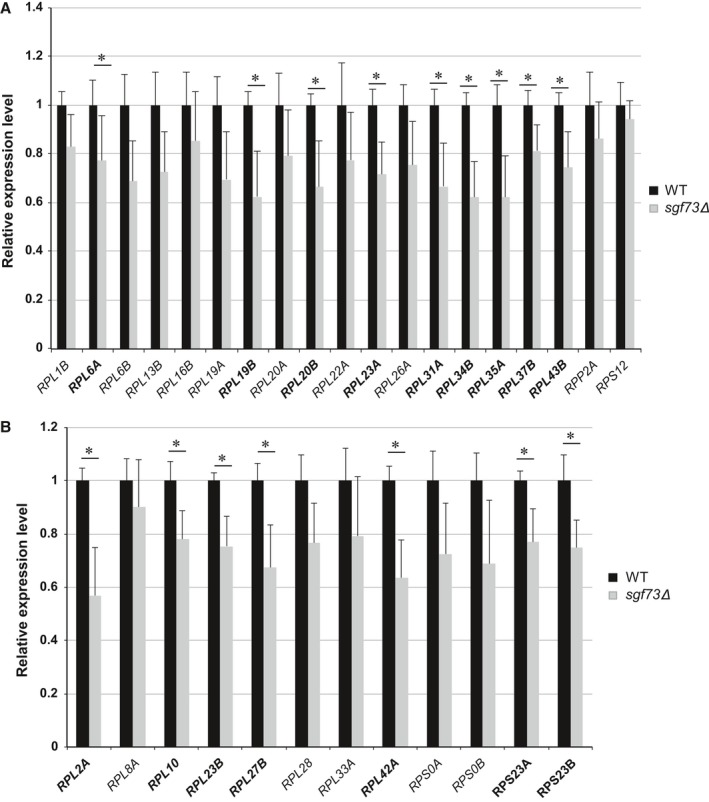
Ribosomal protein subunit genes, subject to Sgf73 occupancy and linked to replicative lifespan extension, display reduced expression in *sgf73Δ* mutants. RNA expression analysis of yeast ribosome protein (RP) subunit genes linked to replicative lifespan extension (A), and of yeast RP subunit genes not linked to replicative lifespan extension (B). Results represent qRT–PCR analysis of indicated RP subunit gene after normalization to the internal control *SCR1*. For each gene, expression in control WT (wild‐type) yeast was arbitrarily set to 1. Bold text indicates significant alterations. *P < 0.05, *t*‐test; *n* = 6 isolates/group; error bars = s.e.m.

To determine whether RP‐encoding genes identified as Sgf73 genomic occupancy sites undergo changes in gene expression upon *SGF73* deletion, we measured RNA expression levels of RP‐encoding genes that promote RLS extension in the *sgf73Δ* mutant. We detected reduced levels of gene expression for all tested RP‐encoding genes, with significant reductions in gene expression documented for nine of these RP‐encoding genes (Fig. 3A). We selected an additional 12 Sgf73‐occupied, non‐RLS‐promoting genes that encode ribosomal subunits for qRT–PCR analysis, as not all RPs may result in significant longevity on their own but could contribute in conjunction with other factors, and we similarly observed reduced expression levels for all 12 RP‐encoding genes, with the majority showing significant reductions (Fig. 3B). Reduced expression of genes encoding ribosomal subunits further supports a role for altered transcriptional regulation of RP‐encoding genes in the dramatic RLS phenotype documented in *sgf73Δ* mutants.

### The *sgf73Δ* mutant exhibits blunted transcriptional repression in response to rapamycin

**Figure 4:**
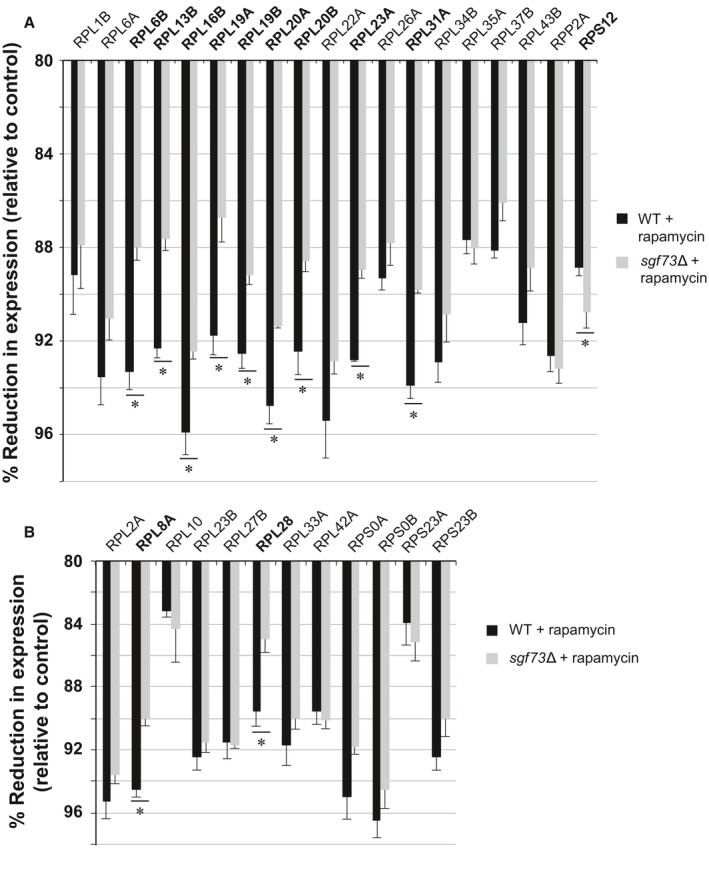
Rapamycin treatment elicits decreased transcriptional repression in *sgf73Δ* mutants. RNA expression analysis of yeast ribosomal protein subunit genes linked to replicative lifespan extension (A), and of yeast RP subunit genes not linked to replicative lifespan extension (B), after rapamycin treatment or exposure to ethanol (control). Results represent qRT–PCR analysis of indicated ribosomal protein subunit gene after normalization to internal control *SCR1*, and then after determining the ratio of expression in rapamycin‐treated yeast to expression in control‐treated yeast. The resultant ratio value was then subtracted from 100% to yield the % reduction in expression. Bold text indicates significant alterations. *P < 0.05, *t*‐test; *n* = 6 isolates/group; error bars = s.e.m.

One well‐documented response to various stress stimuli is a reduction in the transcription of RP‐encoding genes [reviewed in (Xiao & Grove, 2009)]. To test whether downregulation of RP gene transcription is defective upon stress induction in *sgf73Δ* mutants, we treated WT and *sgf73Δ* yeast strains with rapamycin. Rapamycin inhibits TOR, resulting in the repression of RP gene expression, due to a shift toward binding with the repressive Fhl1 cofactor Crf1, as Ifh1 is constitutively bound at RP promoters and its cofactor binding partners determine RP transcriptional levels (Martin *et al*., 2004; Xiao & Grove, 2009). After treatment with 200 ng mL^−1^ rapamycin or ethanol vehicle control, we examined the level of RP gene expression in WT and *sgf73Δ* cells by qRT–PCR analysis. Although all tested RP genes displayed decreased expression upon rapamycin treatment in both WT and *sgf73Δ* yeast, we documented smaller reductions in RP gene expression in *sgf73Δ* strains compared to WT (Fig. 4). Strikingly, nine of the RLS‐linked RP genes (45%) retained significantly higher transcript levels in the *sgf73Δ* mutant in the face of rapamycin stress (Fig. 4A), whereas only two (16%) of the non‐RLS‐linked RP genes exhibited such blunted repression (Fig. 4B). Thus, despite maintaining lower RP gene expression levels in unchallenged conditions, *sgf73Δ* strains did not fully repress RP gene expression when treated with rapamycin. This implies defective RP regulation in both normal and stress conditions in the *sgf73Δ* mutant. Thus, in unchallenged situations, levels are beneficially lower, but during stress, they are higher—allowing cellular processes to persist.

**Figure 5:**
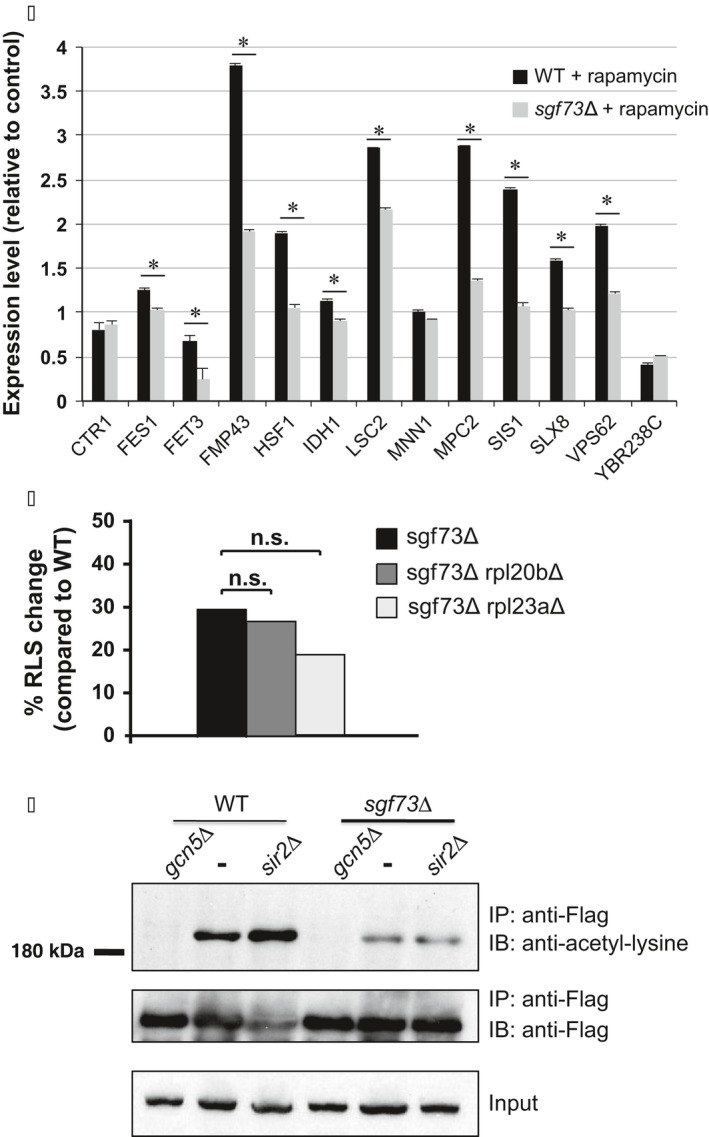
Sgf73 regulation of RLS‐linked RP genes is functionally linked to extreme lifespan extension in *sgf73Δ* yeast. (A) RNA expression analysis of Sgf73‐occupied yeast genes linked to replicative lifespan extension, but not encoding ribosomal protein subunits or protein translation regulators, after rapamycin treatment or exposure to ethanol (control). Results represent qRT–PCR analysis of indicated gene after normalization to the internal control *SCR1*, and then after determining the ratio of expression in rapamycin‐treated yeast to expression in control‐treated yeast. Unlike the response of ribosomal protein subunit genes upon rapamycin treatment, none of these genes exhibited blunted repression in *sgf73Δ* yeast in comparison with WT. *P < 0.05, *t*‐test; *n* = 6 isolates/group; error bars = s.e.m. (B) Double null yeast strains lacking *SGF73* and either *RPL20B* or *RPL23A* were created, and individual yeast cells from each double null strain, from the *sgf73Δ* yeast strain, and from a control wild‐type (WT) strain were subjected to RLS analysis by counting the number of daughter cells per 80 individual yeast cells/strain. Results are shown as the % increase in RLS compared to the WT strain. For *sgf73Δ* vs. *sgf73Δ rpl20bΔ*,* P* = 0.865, *t*‐test. For *sgf73Δ* vs. *sgf73Δ rpl23aΔ*,* P* = 0.765, *t*‐test. (C) Yeast strains were transformed as indicated with Ifh1‐Flag, and then after anti‐Flag antibody immunoprecipitation (IP: anti‐Flag), immunoblots were probed with anti‐acetylated lysine (IB: anti‐acetyl‐lysine). Top: Note increased acetyl‐lysine signal for Ifh1 in *sir2Δ* compared to WT yeast (–), and markedly reduced acetyl‐lysine signal for Ifh1 in *sgf73Δ* yeast without a second deletion mutation (–) compared to WT yeast without a deletion mutation (–). Also, note that the *sgf73Δ sir2Δ* double‐mutant strain does not exhibit increased acetyl‐lysine signal for Ifh1 when compared to the *sgf73Δ* yeast strain, indicating that Sir2‐catalyzed deacetylation of Ifh1 is impaired in the *sgf73Δ* yeast strain. Anti‐Flag immunoblots of anti‐Flag immunoprecipitated material (middle) and of protein lysates from input‐transformed yeast strains (bottom) confirm success of the immunoprecipitation and equivalent expression of transformed Ifh1‐Flag. Yeast lacking *GCN5* acetyltransferase (*gcn5Δ*) were used as a negative control.

To assess whether this transcription dysregulation is a general phenomenon or is restricted to RP‐encoding genes, we quantified the expression levels of non‐RP genes that display Sgf73 occupancy, including genes that promote RLS when deleted. Although significant differences were seen between WT and *sgf73Δ* strains, their expression did not follow the pattern observed for RP genes (Fig. 5A). This suggests that altered RP gene regulation under rapamycin stress conditions is specific to this set of transcripts, and is especially pronounced among RLS‐linked RP genes.

### Sgf73 acts on the same pathway as ribosomal subunit genes to regulate replicative lifespan

Our results indicate that Sgf73 is regulating a subset of RP genes, and loss of Sgf73 regulation of these RP genes accounts for the extreme replicative lifespan phenotype in the *sgf73∆* mutant. To determine whether Sgf73 and RLS‐linked RP genes are acting on the same pathway, we created Sgf73 double null strains with either of two Sgf73‐regulated RLS‐linked RP genes (Rpl20b and Rpl23a) and then performed replicative lifespan analysis in comparison with the corresponding single‐gene null yeast strains. In both cases, we observed no further increase in replicative lifespan (Fig. 5B), indicating that Sgf73 and these RP genes are acting on the same pathway to regulate yeast lifespan.

### Acetylation of Ifh1 is reduced in the *sgf73∆* mutant

We considered the possibility that Sgf73 may affect RP gene expression through Ifh1, which is present at all RP promoters (Cai *et al*., 2013). Ifh1 is acetylated by Gcn5, which affects its binding to Fhl1, thereby modulating RP gene expression (Cherel & Thuriaux, 1995). Opposing activities of SAGA and Sir2 dictate the acetylation status of Ifh1, thereby linking metabolic state to RP gene expression. In cells lacking Gcn5 and the SAGA structural subunit Spt7, Ifh1 acetylation is greatly reduced, whereas disruption of *SIR2* results in higher acetylation levels of Ifh1 (Cai *et al*., 2013; Downey *et al*., 2013). As a member of SAGA, Sgf73 may determine the acetylation levels of Ifh1 by affecting Gcn5 activity or by recruiting the SAGA complex to the regulatory regions of RP genes. Alternatively, Sgf73 may alter Ifh1 acetylation levels through its physical interaction with Sir2 (McCormick *et al*., 2014). To evaluate the role of Ifh1 acetylation in Sgf73 mutants, we immunoprecipitated Ifh1‐Flag from WT and *sgf73∆* strains and immunoblotted with an anti‐acetyl‐lysine antibody. We observed reduced Ifh1 acetylation levels in the *sgf73∆* strain (Fig. 5C), although global histone H3K9/14 acetylation levels were not altered in the *sgf73∆* cells (Fig. S5), as previously reported (Lee *et al*., 2009). This lack of global disruption of SAGA target acetylation in the *sgf73∆* strain indicates that loss of Sgf73 function specifically affects SAGA‐mediated acetylation of Ifh1, resulting in increased Ifh1 deacetylation. When *SIR2* function is lost, acetylation of Ifh1 markedly increases (Fig. 5C), consistent with Sir2 deacetylation of Ifh1, as shown previously (Downey *et al*., 2013). To determine whether deletion of *SIR2* leads to increased Ifh1 acetylation levels in the *sgf73∆* mutant, we constructed the *sgf73∆ sir2∆* double mutant and measured Ifh1 acetylation. Strikingly, Ifh1 acetylation levels were not higher in the *sgf73∆ sir2∆* double mutant, compared to the *sgf73∆* mutant alone (Fig. 5C). These results indicate that Sir2‐dependent deacetylation of Ifh1 is already impaired in the absence of Sgf73, suggesting that Sgf73 is important for Sir2 targeting, an observation consistent with our previous findings (McCormick *et al*., 2014). When we compared Ifh1 acetylation in the *sgf73∆ sir2∆* double mutant to the *sir2∆* mutant alone, we observed reduced Ifh1 acetylation, indicating that a defect in SAGA complex‐mediated acetylation of Ifh1 is present in the *sgf73∆* mutant. Taken together, these results demonstrate that proper acetylation regulation of Ifh1 is lost in the *sgf73∆* strain through a disruption of both normal Gcn5 acetylation and Sir2 deacetylation.

## Discussion

Sgf73 is a transcriptional adaptor linking the core of the SAGA transcriptional coactivator complex to the Ubp8 DUB module. Previous studies have highlighted the importance of Sgf73 for Ubp8 DUB activity, showing that deletion of *SGF73* is sufficient to completely abolish Ubp8‐regulated deubiquitination of histone H2BK123 (Kohler *et al*., 2008). Furthermore, we have documented an interaction between Sgf73 and the deacetylase Sir2 and demonstrated that *sgf73Δ* cells have enhanced Sir2‐dependent activity (McCormick *et al*., 2014). These findings implicate Sgf73 as a coordinator of gene regulation between the Sir2 and Ubp8 chromatin modifiers, which is key to lifespan extension in *sgf73Δ* cells. In this study, we sought to identify the genomic regions bound by Sgf73 and thus determine how Sgf73 may coordinate epigenetic modifications to modulate gene expression in order to gain additional insight into mechanisms of aging and the human neurodegenerative disease SCA7.

Deletion of *SGF73* results in dramatic RLS extension, increasing median RLS by 65% and maximum RLS by 53% (McCormick *et al*., 2014). Our previous studies indicated that two major contributing factors to extended RLS in *sgf73Δ* mutants are increased H2BK123 ubiquitination and altered Sir2‐dependent deacetylation. In this study, we asked whether transcription dysregulation may also directly contribute to RLS extension in *sgf73* mutants. To define mechanisms of Sgf73 loss‐of‐function RLS extension, we performed Sgf73 ChIP‐Seq and identified 388 unique chromatin regions of Sgf73 occupancy. Most striking was the enriched occupancy of Sgf73 at positions 5′ to ribosomal subunit genes (57 genes; *P* = 1.96e‐34). Among these genes, 20 are known to extend RLS upon their deletion. In total, we found Sgf73 occupancy 5′ of 33 genes known to extend RLS upon deletion, including 20 ribosomal‐related genes.

With the finding of enrichment for Sgf73 occupancy at ribosomal genes and deep connections between ribosomal gene deletion and RLS extension, we asked whether loss of Sgf73 elicited transcriptional changes in these genes, and documented reduced RP gene expression upon *SGF73* deletion. We then constructed double null yeast strains lacking *SGF73* and either of two Sgf73‐regulated RLS‐linked RP genes and observed no further extension in RLS in such double null strains. Thus, Sgf73 appears to have a previously unrecognized role in ribosome biogenesis, and impaired ribosome biogenesis, resulting in reduced protein translation, is likely a major contributor to the extreme RLS extension observed in the *sgf73Δ* mutant. As reduced protein translation has been shown to promote lifespan extension in mice (Selman *et al*., 2009), our findings reinforce the importance of protein production balance as a deeply conserved factor in determining organismal lifespan.

A role for Sgf73 in ribosomal biogenesis is perhaps not unexpected, as SAGA is a stress‐induced transcriptional coactivator complex (Huisinga & Pugh, 2004), and previous studies have identified SAGA binding at RP genes (Cai *et al*., 2011), as well as binding of Gcn5 at RP genes (Venters *et al*., 2011). Gcn5 acetylates the RP gene transcription coactivator Ifh1 to reduce RP gene expression, and Ifh1 is also subject to Sir2 deacetylation (Cai *et al*., 2013; Downey *et al*., 2013). Indeed, Sgf73 appears to play a role in ribosomal biogenesis under treatment with rapamycin, a TOR inhibitor. Upon treatment with rapamycin, RP gene expression is reduced in WT cells through altered TOR regulation of Ifh1. Although we also observed reduced RP gene expression in *sgf73Δ* cells, this was not to the same extent as in WT cells, identifying Sgf73 as a key target of Ifh1 regulation. Our previous studies identified Sir2 as a key player in *sgf73Δ* RLS extension and documented a physical interaction between Sgf73 and Sir2 (McCormick *et al*., 2014). As ribosome protein biogenesis is highly dependent on Ifh1, with its recruitment and acetylation state affecting the degree to which RP genes are expressed (Downey *et al*., 2013), we considered a role for altered Ifh1 acetylation in the transcriptional dysregulation of RP‐encoding genes in the *sgf73Δ* mutant. Both SAGA and Sir2 influence acetylation of Ifh1, with Gcn5 promoting acetylation and Sir2 mediating deacetylation (Downey *et al*., 2013). Current models of RP gene expression regulation posit that a cycle of rapid acetylation and deacetylation of Ifh1 permits a burst of RP gene expression when nutrients become plentiful. According to this model, Sir2‐mediated Ifh1 deacetylation resets the system so that increased RP gene transcription can occur, and this increased transcription is then restrained upon Ifh1 acetylation (Downey *et al*., 2013). When we examined the acetylation levels of Ifh1 in the *sgf73Δ* mutant, we observed reduced Ifh1 acetylation. In the *sgf73Δ sir2Δ* double‐mutant strain, we found that Ifh1 acetylation levels were comparable to the *sgf73Δ* mutant, but were reduced compared to the *sir2Δ* mutant strain, indicating that both SAGA acetylation and Sir2 deacetylation of Ifh1 are impaired in the *sgf73Δ* strain. Thus, in *sgf73Δ* mutants, dynamic regulation of Ifh1 acetylation is lost, resulting in reduced RP gene expression at baseline and a failure to properly repress RP gene expression under stress. These findings support a model whereby Sgf73 is necessary for Sir2 deacetylation of Ifh1, and Sgf73 is also required for optimal Gcn5 acetylation of Ifh1. Reduced ribosomal biogenesis during progressive aging in *sgf73Δ* mutants is likely a factor in the extended RLS, as reduction in 60S ribosome subunits extends RLS. Taken together, these results highlight altered acetylation dynamics of target proteins as an upstream event in transcriptional dysregulation of ribosome biogenesis, which in the case of yeast aging appears to be beneficial. Whether ataxin‐7, the mammalian orthologue of Sgf73, is similarly important for acetylation‐mediated regulation of transcription and how polyglutamine expansion of ataxin‐7 in the human neurological disorder SCA7 may alter this acetylation regulation to promote neurodegeneration are important questions for future studies.

## Materials and methods

### Yeast strain construction and dilution assay

Tagging was performed as described, using the pFA6a‐13‐Myc‐kanMX6 plasmid and C‐terminal tagging primers (Longtine *et al*., 1998). In brief, the 13‐Myc‐KanMX6 sequence was amplified using primers with overhangs corresponding to 50 bp 5′ to the *SGF73* or *UBP8* stop codon, as well as 50 bp 3′ to the stop codon. Competent WT cells were prepared and transformed with the amplified product to integrate the tag by homologous recombination. Positive clones were selected on G418. Molecular genotyping was used to confirm the tags’ presence; positive strains were backcrossed to WT cells. Cells were grown overnight in 3 mL of YPD, then 1 A_600_ equivalent of cells was spun down, spent media were removed, and cells were resuspended in 1 mL of sterile water. 1:5 serial dilutions were then pinned onto control, selective, and/or drug plates.

### Chromatin immunoprecipitation

A 5 mL of YPD culture from a single yeast colony grown overnight at 30 °C was diluted in 100 mL of YPD for harvest at ~A_600_ L mL^−1^. Cells were fixed with 0.86% formaldehyde at room temperature (RT) for 25 min. The reaction was quenched with 125 mm glycine and 0.2% NH_4_OH for 5 min at RT. Cells were collected by centrifugation for 15 min. The pellet was washed three times with 20 mL of cold phosphate‐buffered saline (PBS). Washed cells were resuspended in the remaining wash buffer, moved to a 2‐mL tube, spun, and additional liquid removed. Cells were lysed in 1.5 mL of FA‐lysis buffer (50 mm HEPES‐KOH pH 7.5, 140 mm NaCl, 1 mm EDTA pH 8.0 0.1% sodium deoxycholate, 1% Triton X‐100, and 1x Roche complete proteinase inhibitors) with additional detergents (0.5% NP‐40 and 0.1% SDS) and divided between three 1.5‐mL tubes. Acid‐washed glass beads were then added to the meniscus, and cells were lysed by bead beating at 4 °C for 1 h. Lysate was transferred to a new 1.5‐mL tube. Chromatin was then sheared in a precooled horn sonicator (Sonic Dismembrator FB‐505, Fisher Scientific, Waltham, Massachusetts, USA), using pulse cycles of 20 s on and 30 s off at 80% amp for a total time of 30 min to achieve fragments of 250–400 bp. After sonication, tubes were spun at 4 °C for 5 min and the supernatant was transferred to low‐bind tubes (Lifetech, Carlsbad, California, USA AM12450). An equal volume of FA‐lysis buffer was added, and lysate was precleared with 10 μL of protein A:G magnetic beads for 30 min at 4 °C. A sample from each preparation was taken after preclearing to analyze input chromatin and protein. 100 μL of anti‐c‐Myc EZview resin (Sigma, St. Louis, Missouri, USA E6654‐1ML) was added to the samples and rotated overnight at 4 °C. Postbinding beads were washed twice with 1 mL FA‐lysis buffer, twice with 1 mL of wash buffer (10 mm Tris pH 8, 0.25M LiCl, 0.5% NP‐40, 0.5% sodium deoxycholate, 1 mm EDTA), and once with 1 mL of Tris‐EDTA (TE). Samples were de‐cross‐linked with 100 μL of TE + 1% SDS (with 10 μL taken for protein analysis) and incubated at 65 °C for 6 h. Following cross‐link reversal, 8 μL of TE and 2 μL of RNase were added and incubated at 37 °C for 1 h. 1.5 μL of proteinase K (20 mg mL^−1^) was added followed by overnight incubation at 37 °C. DNA was purified using the MinElute PCR Kit (Qiagen, Hilden, Germany 28006) and eluted with 13 μL of nuclease‐free water. Fragment size was analyzed on 6% acrylamide gels.

### ChIP‐Seq analysis

Libraries for sequencing were generated using the ChIP'd fragments and the Ovation ultralow DR multiplex 1–8 library system (NuGEN—0330). Library amplification was performed using 18 cycles of 94 °C—30 s, 60 °C—30 s, 72 °C—1 min, with a 2‐min 72 °C predenaturation step and 5‐min 72 °C extension step added. After library construction, fragments were size‐selected. Libraries were separated on 6% TBE‐acrylamide using Invitrogen's 25‐bp ladder, and DNA in the 150‐ to 400‐bp range was excised. DNA was recovered in 350 μL of elution buffer (300 mm NaCl, 10 mm Tris pH 7.6). The mixture was transferred to a SpinX tube (Sigma CLS1862) to remove gel from the DNA in suspension. DNA was precipitated in 1 mL cold 100% ethanol, 35 μL of sodium acetate (3M pH 5.2), and 2 μL glycogen (5 mg mL^−1^). Libraries were resuspended in 15 μL of Qiagen EB, and 1 μL was run on a 2100 Bioanalyzer DNA 100 chip (Agilent, Santa Clara, California, USA 5067‐1504). Sequencing was performed at Beijing Genomics Institute on the Illumina Hi‐seq 2000 platform. Single‐end 50‐bp read sequencing of the pooled libraries. Postsequencing, all reads were sorted by barcode and low‐quality sequence reads were removed. All ChIP‐Seq data are deposited and publicly available via Gene Expression Omnibus accession #GSE76461.

### Bioinformatics analysis and validation

To analyze the sequencing reads, we employed homer (Hypergeometric Optimization of Motif EnRichment) v4.2 http://biowhat.ucsd.edu/homer/. Raw sequence reads were evaluated with fastqc (http://www.bioinformatics.babraham.ac.uk/projects/fastqc/) for quality control. Sequence duplication was compensated for using only uniquely mapping reads. The *S. cerevisiae* S288c genome assembly from the Saccharomyces Genome Database (GCA_000146055.2) was used for all alignments and annotations. Oligonucleotide sequences for evaluation of ChIP and validation of occupancy will be furnished upon request.

### Whole‐cell lysate preparation and anti‐Flag immunoprecipitations

Protocol was adapted from Downey *et al*., 2013. Between 90 and 100 A_600_ equivalents of cells were collected by centrifugation, washed with phosphate‐buffered saline, and stored at −80 °C until lysis. Pellets were resuspended in 500 μL cold lysis buffer (0.5% Triton, 200 mm NaCl, 50 mm Tris–HCl pH 7.5, and 1 mm EDTA), supplemented with 10 mm sodium butyrate, 10 mm nicotinamide, 5 mm sodium fluoride, 1 mm dithiothreitol, and a cocktail of protease inhibitors including phenylmethylsulfonyl fluoride, leupeptin, benzamidine HCl, pepstatin, and tosyl phenylalanyl chloromethyl ketone (lysis buffer supplemented or LBS). Glass lysis beads were added to the meniscus in 50‐mL conical tubes and lysed at 4 °C. Six 1‐min durations of vortexing were carried out with 1 min on ice in between. Lysates were recovered by pipette, and the beads were washed with LBS. This wash was combined with the lysate. Extracts were clarified by centrifugation at 4 °C. Anti‐Flag M2 beads (Sigma‐Aldrich, St. Louis, Missouri, USA) were added to the lysate and incubated for 2–3 h on cold room nutator. The beads were washed three times with LBS. Following washes, remaining liquid was aspirated with an 18‐gauge needle. Immunoprecipitated proteins were eluted with 100 μL 2xUSB (8 M urea, 4% SDS, 10% ß‐mercaptoethanol, 125 mm Tris, pH 6.8) at 65 °C for 10 min. Prior to loading onto an 8% SDS‐PAGE gel, eluates were boiled for 5 min. Typically 50% of immunoprecipitated material was analyzed for each anti‐acetyl‐lysine (Chemicon, Temecula, California, USA) immunoblot.

## Conflict of interest

None declared.

## Funding

This study was supported by funds from the NIH: R01 EY014061 and R01 AG033082 (A.R.L.S.), R01 GM090177 (L.P.), T32 GM008666 (A.G.M.), T32 AG000266 (M.A.M.), and F32 GM089101 (R.M.G.).

## Supporting information


**Fig. S1** Validation of C‐terminal Myc‐tagged Sgf73 constructs and Myc ChIP.

**Fig. S2** Confirmation of Sgf73‐Myc function and Sgf73/Ubp8 ChIP for expected occupancy sites.

**Fig. S3** Gene ontology analysis of Sgf73 ChIP‐Seq data and comparison of Sgf73‐occupied genes with replicative lifespan promoting genes.

**Fig. S4** Ubp8 shares occupancy with Sgf73 RLS‐linked RP peaks.

**Fig. S5** Overall acetylation activity is not diminished in *sgf73Δ* yeast.

**Table S1** Sgf73 occupancy peaks from ChIP‐Seq analysis

**Table S2** Sgf73 and Ubp8 shared occupancy peaks from ChIP‐Seq analysis
