# Supplementary figures and images for: The replicative lifespan‐extending deletion of SGF73 results in altered ribosomal gene expression in yeast
Source: Aging Cell. 2017 May 31;16(4):785–96. doi: 10.1111/acel.12611 (PMC5506417; doi:10.1111/acel.12611)

**A**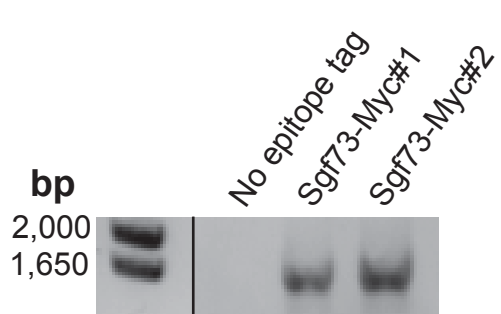**B**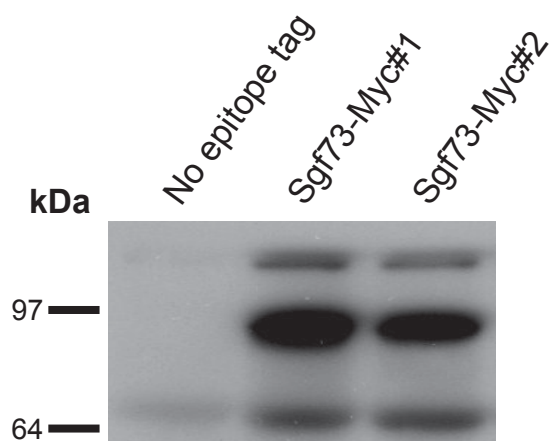**C**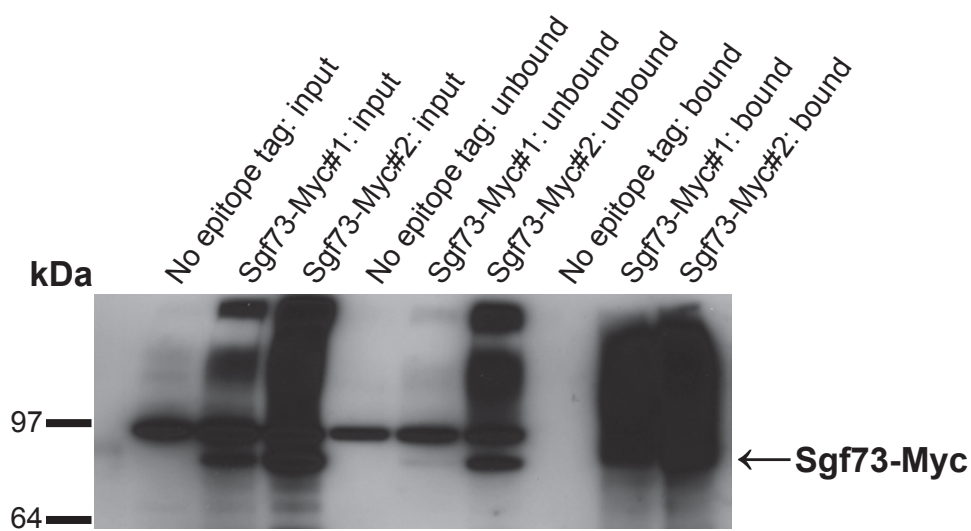

Supplement: Supplementary file 1 — Fig. S1 Validation of C‐terminal Myc‐tagged Sgf73 constructs and Myc ChIP. [file ACEL-16-785-s001.pdf]

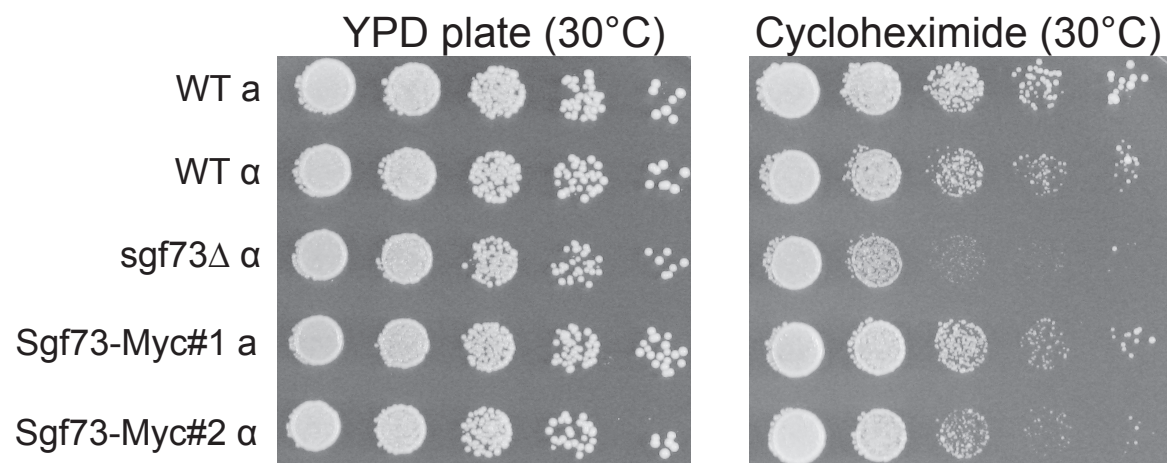

Supplement: Supplementary file 2 — Fig. S2 Confirmation of Sgf73‐Myc function and Sgf73/Ubp8 ChIP for expected occupancy sites. [file ACEL-16-785-s002.pdf]

A

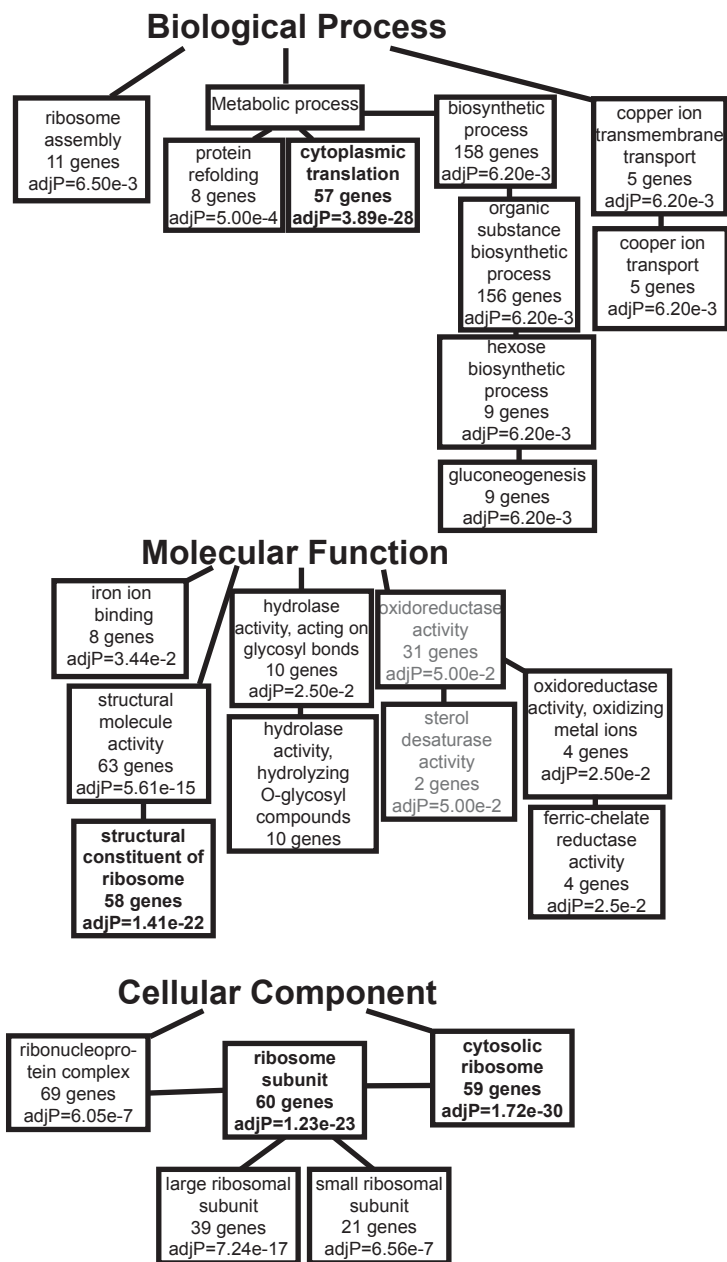

B

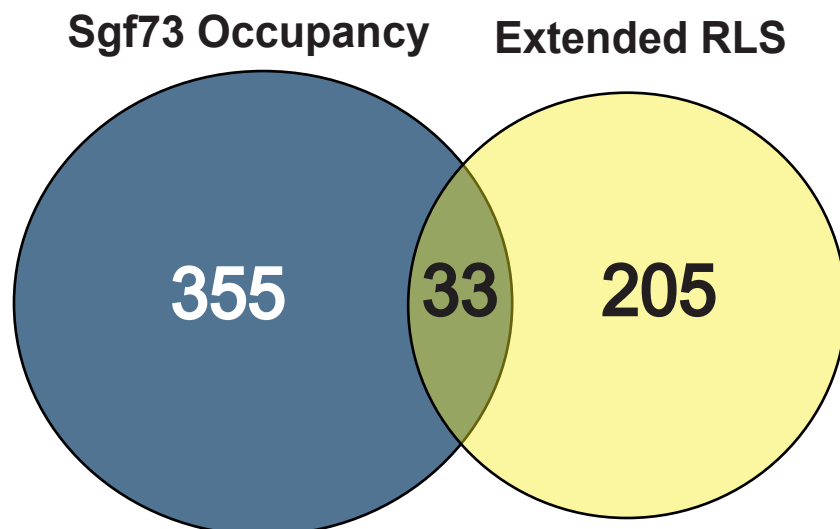

Supplement: Supplementary file 3 — Fig. S3 Gene ontology analysis of Sgf73 ChIP‐Seq data and comparison of Sgf73‐occupied genes with replicative lifespan promoting genes. [file ACEL-16-785-s003.pdf]

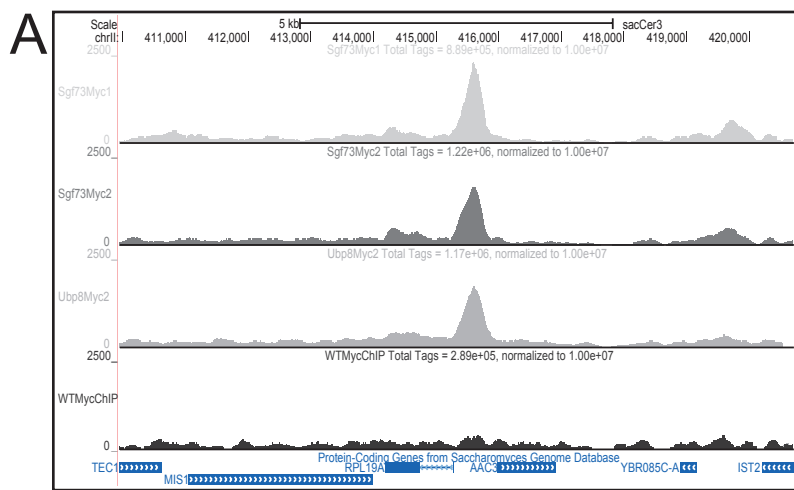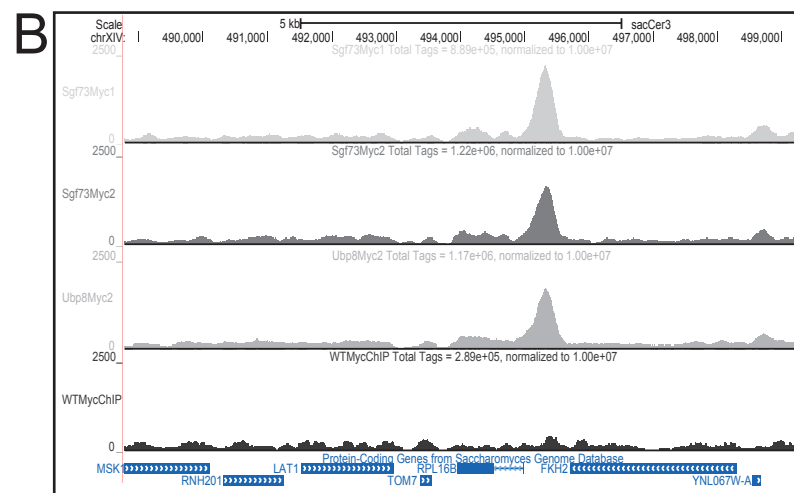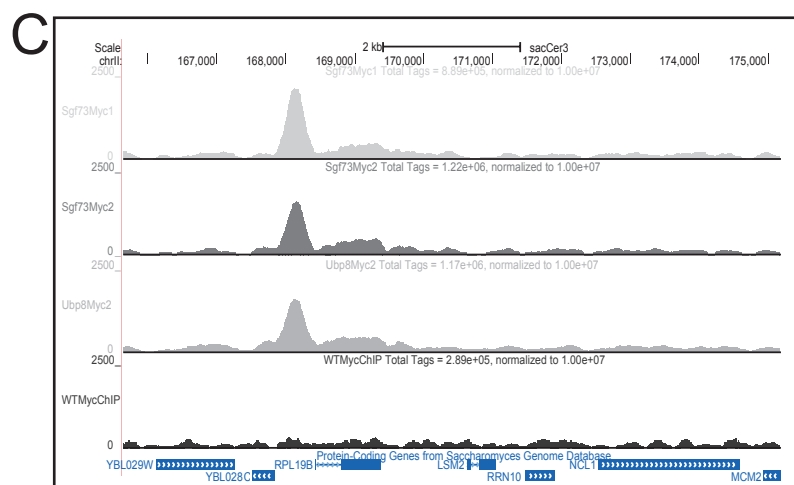

Supplement: Supplementary file 4 — Fig. S4 Ubp8 shares occupancy with Sgf73 RLS‐linked RP peaks. [file ACEL-16-785-s004.pdf]

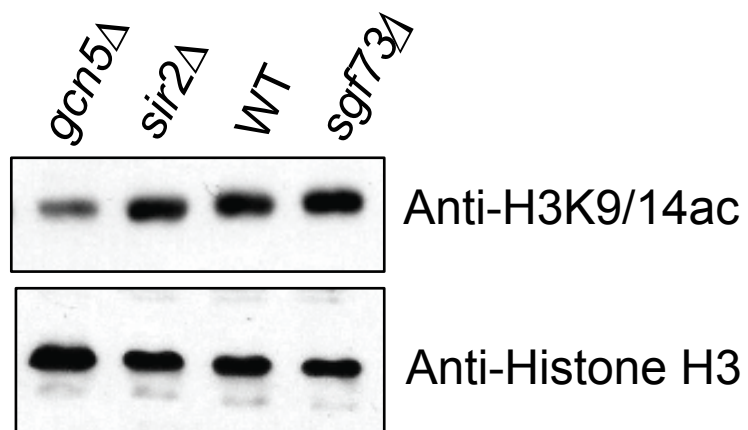

Supplement: Supplementary file 5 — Fig. S5 Overall acetylation activity is not diminished in sgf73Δ yeast. [file ACEL-16-785-s005.pdf]
