# Supplementary material for: The replicative lifespan‐extending deletion of SGF73 results in altered ribosomal gene expression in yeast
Source: Aging Cell. 2017 May 31;16(4):785–96. doi: 10.1111/acel.12611 (PMC5506417; doi:10.1111/acel.12611)
Supplement: Supplementary file 6 — Table S1 Sgf73 occupancy peaks from ChIP‐Seq analysis [file ACEL-16-785-s006.pdf]

Table S1. Sgf73 occupancy peaks from ChIP-Seq analysis

Chr #	Start	End	Peak Score	Distance:TSS	Nearest PromoterI	Gene Name	Gene Description
chrI	141707	141838	3693	-341	YAL005C	SSA1	Hsp70 family ATPase SSA1
chrXVI	645455	645577	3649.449951	-434	YPR036W-A	YPR036W-A	hypothetical protein
chrXVI	75080	75186	3532.5	-414	YPL250C	ICY2	Icy2p
chrIV	465299	465407	3402.100098	30	YDR010C		
chrVII	772109	772212	3341.25	-294	YGR142W	BTN2	Btn2p
chrIII	137335	137440	3245.25	-359	YCR012W	PGK1	phosphoglycerate kinase
chrV	69152	69273	3229.25	53	YEL045C		
chrXII	97654	97755	3146.049805	-219	YLL024C	SSA2	Hsp70 family chaperone SSA2
chrVII	914977	915084	3095.399902	-211	YGR211W	ZPR1	Zpr1p
chrIV	416997	417100	3093.399902	-340	YDL020C	RPN4	Rpn4p
chrXII	370597	370697	2938.949951	-144	YLR112W		
chrIV	1490147	1490262	2916.050049	-214	YDR524C-A		
chrXIII	388438	388553	2899.699951	-335	YMR058W	FET3	Ferro-O2-oxidoreductase
chrXIV	619847	619948	2890.5	-170	YNL006W	LST8	Lst8p
chrII	477329	477444	2832.100098	-285	YBR118W	TEF2	Tef2p
chrVII	371312	371427	2816.899902	-7	YGL072C		
chrVII	483464	483589	2808.100098	266	YGL007W		
chrIX	249160	249275	2802.5	-774	YIL056W	VHR1	Vhr1p
chrXII	368368	368489	2778.350098	-304	YLR108C	YLR108C	hypothetical protein
chrXV	619323	619443	2765.75	-457	YOR153W	PDR5	ATP-binding cassette multidrug transporter PDR5
chrXI	518624	518748	2752.850098	-120	YKR040C		
chrXV	83246	83361	2713.899902	-383	YOL126C	MDH2	malate dehydrogenase MDH2

chrXVI	76423	76536	2697.600098	-240	YPL249C-A	RPL36B	ribosomal 60S subunit protein L36B
chrIX	386713	386823	2683.350098	-1067	YIR018C-A	YIR018C-A	hypothetical protein
chrVII	884045	884150	2639.049805	-287	YGR192C	TDH3	glyceraldehyde-3-phosphate dehydrogenase (phosphorylating) TDH3
chrXV	797080	797207	2588.899902	-350	YOR246C	ENV9	Env9p
chrVII	609975	610076	2582.850098	-539	YGR060W	ERG25	methylsterol monooxygenase
chrVIII	122344	122456	2534.450195	365	YHR007C-A	YHR007C-A	hypothetical protein
chrII	444793	444899	2486.050049	-153	YBR101C	FES1	Fes1p
chrXV	877838	877959	2474.350098	-213	YOR298C-A	MBF1	Mbf1p
chrXII	86985	87090	2469.25	-366	YLL027W	ISA1	Isa1p
chrXIII	306644	306749	2452.200195	-793	YMR017W	SPO20	Spo20p
chrXIV	87501	87612	2443.75	-341	YNL289W	PCL1	Pcl1p
chrVII	785546	785668	2433.800049	-170	YGR146C-A	YGR146C-A	hypothetical protein
chrXIII	632111	632218	2425.75	-191	YMR186W	HSC82	Hsp90 family chaperone HSC82
chrXVI	641341	641530	2407.850098	-773	YPR035W	GLN1	glutamate--ammonia ligase
chrIV	132841	132988	2401.200195	-523	YDL182W	LYS20	homocitrate synthase LYS20
chrVI	221769	221872	2330.100098	-402	YFR031C-A	RPL2A	ribosomal 60S subunit protein L2A
chrVII	809750	809868	2316.850098	-389	YGR161C	RTS3	Rts3p
chrIV	1080572	1080682	2313.25	-428	YDR309C	GIC2	Gic2p
chrIII	78427	78535	2311.699951	-562	YCL025C	AGP1	Agp1p
chrXVI	679114	679269	2307.100098	240	YPR064W		
chrXIV	739430	739557	2297.049805	-458	YNR060W	FRE4	Fre4p
chrXVI	40478	40599	2293.699951	-505	YPL265W	DIP5	Dip5p
chrIV	765359	765481	2291.649902	-265	YDR151C	CTH1	Cth1p

chrVI	225173	225280	2282.100098	-457	YFR033C	QCR6	ubiquinol--cytochrome-c reductase subunit 6
chrXV	109715	109850	2261.600098	-515	YOL109W	ZEO1	Zeo1p
chrXI	99841	99943	2238.050049	-779	YKL182W	FAS1	tetrafunctional fatty acid synthase subunit FAS1
chrXVI	785815	785935	2232	-333	YPR124W	CTR1	Ctr1p
chrXI	381883	381993	2218.75	-80	YKL032C	IXR1	Ixr1p
chrVII	310724	310830	2204.850098	-190	YGL103W	RPL28	ribosomal 60S subunit protein L28
chrXII	253425	253546	2199.549805	-376	YLR056W	ERG3	C-5 sterol desaturase
chrIV	806329	806433	2185.649902	-240	YDR171W	HSP42	Hsp42p
chrX	521723	521839	2181.350098	-179	YJR045C	SSC1	Hsp70 family ATPase SSC1
chrXI	258357	258458	2181.050049	506	YKL097C		
chrXVI	98796	98904	2175.449951	-225	YPL240C	HSP82	Hsp90 family chaperone HSP82
chrXIII	551398	551502	2158.399902	-243	YMR142C	RPL13B	ribosomal 60S subunit protein L13B
chrXIII	362278	362398	2112.649902	289	YMR045C	YMR045C	gag-pol fusion protein
chrXIII	302883	303024	2109.600098	-468	YMR015C	ERG5	C-22 sterol desaturase
chrV	306028	306150	2074.899902	-234	YER074W	RPS24A	ribosomal 40S subunit protein S24A
chrIII	122610	122748	2055.049805	324	YCR006C		
chrI	71087	71225	2043.899902	-630	YAL038W	CDC19	pyruvate kinase CDC19
chrIV	221994	222116	2043.699951	-254	YDL133C-A	RPL41B	ribosomal 60S subunit protein L41B
chrXIII	915350	915470	2029.850098	-872	YMR319C	FET4	Fet4p
chrII	140573	140678	2027.449951	-365	YBL042C	FUI1	Fui1p
chrII	415546	415648	2027.050049	-336	YBR084C-A	RPL19A	ribosomal 60S subunit protein L19A
chrV	225532	225632	2020	-307	YER037W	PHM8	Phm8p

chrVII	598973	599078	2016.849976	-392	YGR055W	MUP1	Mup1p
chrIV	976598	976701	1996.599976	580	YDR260C	SWM1	Swm1p
chrII	221556	221691	1995.699951	293	YBL005W-B	YBL005W-B	gag-pol fusion protein
chrVII	726667	726769	1994.349976	-239	YGR117C	YGR117C	hypothetical protein
chrIV	894079	894197	1987.550049	364	YDR215C		
chrII	332549	332653	1984.5	-230	YBR048W	RPS11B	ribosomal 40S subunit protein S11B
chrXIV	495272	495401	1984.149902	-335	YNL069C	RPL16B	ribosomal 60S subunit protein L16B
chrXI	164629	164731	1972.899902	-295	YKL152C	GPM1	phosphoglycerate mutase GPM1
chrIII	228865	228974	1965.25	-391	YCR065W	HCM1	Hcm1p
chrII	168066	168211	1955	-285	YBL027W	RPL19B	ribosomal 60S subunit protein L19B
chrXIII	253438	253555	1952.350098	-224	YML007C-A	YML007C-A	hypothetical protein
chrIV	591702	591833	1930	-423	YDR072C	IPT1	inositolphosphotransferase
chrXIII	408541	408658	1925.349976	-555	YMR070W	MOT3	Mot3p
chrXIV	576314	576456	1920.25	-335	YNL031C	HHT2	Hht2p
chrXII	645883	646012	1898.299927	6	YLR255C		
chrVII	149217	149324	1897.75	246	YGL188C		
chrXIV	63173	63298	1897.200073	-292	YNL302C	RPS19B	ribosomal 40S subunit protein S19B
chrXIII	225563	225673	1892.600098	-253	YML025C	YML6	mitochondrial 54S ribosomal protein YmL6
chrXII	809351	809459	1890.649902	-592	YLR342W	FKS1	Fks1p
chrXVI	453531	453639	1888.549927	150	YPL056C	LCL1	Lcl1p
chrVII	23427	23542	1886.400024	-451	YGL253W	HXK2	hexokinase 2
chrXII	1019008	1019117	1878.75	-154	YLR441C	RPS1A	ribosomal 40S subunit protein S1A
chrVI	106456	106562	1876.299927	94	YFL015W-A		

chrIV	769194	769295	1871.199951	-244	YDR155C	CPR1	peptidylprolyl isomerase CPR1
chrIV	1468880	1469013	1857.5	-454	YDR510W	SMT3	SUMO family protein SMT3
chrXII	88325	88433	1855.5	-244	YLL026W	HSP104	chaperone ATPase HSP104
chrXV	987633	987812	1826.649902	1060	YOR348C	PUT4	Put4p
chrXII	568200	568307	1820.25	-314	YLR214W	FRE1	Fre1p
chrVII	254368	254492	1819.050049	-211	YGL135W	RPL1B	ribosomal 60S subunit protein L1B
chrXV	968087	968210	1809.400024	326	YOR343C		
chrXI	327452	327569	1799.650024	-23	YKL060C	FBA1	fructose-bisphosphate aldolase FBA1
chrXVI	432008	432169	1799.650024	193	YPL062W		
chrXIII	540470	540591	1797.449951	-474	YMR135C	GID8	glucose-induced degradation complex subunit GID8
chrIX	254323	254427	1793.899902	-168	YIL054W	YIL054W	hypothetical protein
chrVIII	48291	48430	1792.599976	-392	YHL029C	OCA5	Oca5p
chrXV	27930	28050	1785.900024	906	YOL155W-A	YOL155W-A	hypothetical protein
chrXVI	794676	794786	1764.150024	-233	YPR131C	NAT3	Nat3p
chrIV	892501	892609	1760.400024	-320	YDR214W	AHA1	Aha1p
chrIV	1165017	1165208	1759.149902	-452	YDR345C	HXT3	Hxt3p
chrX	291143	291259	1751.350098	-427	YJL079C	PRY1	Pry1p
chrII	408680	408781	1741.449951	-439	YBR083W	TEC1	Tec1p
chrIV	974377	974480	1740.800049	-185	YDR258C	HSP78	chaperone ATPase HSP78
chrIV	321879	321984	1733.550049	-295	YDL075W	RPL31A	ribosomal 60S subunit protein L31A
chrIV	550979	551096	1732.649902	-461	YDR046C	BAP3	Bap3p
chrXIV	559344	559470	1728.550049	-405	YNL037C	IDH1	isocitrate dehydrogenase (NAD(+)) IDH1
chrXI	67856	67969	1724.5	-446	YKL201C	MNN4	Mnn4p

chrIV	357221	357333	1721.399902	-518	YDL055C	PSA1	mannose-1-phosphate guanylyltransferase
chrXII	737858	737998	1715.099976	-380	YLR304C	ACO1	aconitate hydratase ACO1
chrXVI	653977	654090	1714.699951	-133	YPR043W	RPL43A	ribosomal 60S subunit protein L43A
chrXV	253915	254041	1710.600098	-319	YOL039W	RPP2A	ribosomal protein P2A
chrVII	516402	516507	1696.400024	-489	YGR014W	MSB2	Msb2p
chrV	396441	396542	1692.199951	-278	YER117W	RPL23B	ribosomal 60S subunit protein L23B
chrIX	257361	257495	1688.699951	-365	YIL052C	RPL34B	ribosomal 60S subunit protein L34B
chrV	364279	364401	1678	-249	YER103W	SSA4	Hsp70 family chaperone SSA4
chrXII	241894	241996	1676	-287	YLR048W	RPS0B	ribosomal 40S subunit protein S0B
chrXIII	880621	880722	1673.449951	-439	YMR305C	SCW10	Scw10p
chrXIV	444493	444598	1658.099976	-230	YNL096C	RPS7B	ribosomal 40S subunit protein S7B
chrXIV	331036	331137	1652.699951	-236	YNL162W	RPL42A	ribosomal 60S subunit protein L42A
chrXIII	661832	661940	1646.300049	-758	YMR199W	CLN1	Cln1p
chrXV	232167	232269	1644.25	-463	YOL052C-A	DDR2	Ddr2p
chrVII	920127	920250	1631.900024	-387	YGR214W	RPS0A	ribosomal 40S subunit protein S0A
chrX	651426	651538	1628.449951	-419	YJR123W	RPS5	Rps5p
chrXIII	124367	124483	1627.349976	-253	YML073C	RPL6A	ribosomal 60S subunit protein L6A
chrXV	779633	779774	1625.900024	-167	YOR235W		
chrX	157391	157495	1625.800049	-170	YJL136C	RPS21B	Rps21bp

chrXII	837671	837775	1623.75	-366	YLR354C	TAL1	sedoheptulose-7-phosphate:D-glyceraldehyde-3-phosphate transaldolase TAL1
chrVII	557749	557863	1622.199951	241	YGR035W-A	YGR035W-A	hypothetical protein
chrX	75554	75706	1620.550049	-303	YJL189W	RPL39	Rpl39p
chrIV	491198	491321	1614.149902	242	YDR024W		
chrXIII	26153	26277	1612.949951	203	YML122C		
chrV	141172	141289	1601.400024	-412	YEL008C-A		
chrXII	941022	941126	1596.400024	-409	YLR410W-A	YLR410W-A	gag protein
chrXII	1012184	1012302	1595.599976	-220	YLR437C	DIF1	Dif1p
chrVII	398060	398174	1594.449951	-499	YGL056C	SDS23	Sds23p
chrXVI	297165	297293	1591.349976	-324	YPL135W	ISU1	Isu1p
chrXIV	481044	481146	1584.050049	-296	YNL077W	APJ1	Apj1p
chrIII	17042	17170	1578.25	-184	YCL063W	VAC17	Vac17p
chrVII	438598	438715	1571.650024	-435	YGL030W	RPL30	ribosomal 60S subunit protein L30
chrIX	68403	68520	1566	-247	YIL148W	RPL40A	ubiquitin-ribosomal 60S subunit protein L40A fusion protein
chrIV	234460	234589	1565.099976	-403	YDL127W	PCL2	Pcl2p
chrXII	704439	704551	1564.5	186	YLR279W		
chrX	236586	236708	1561.900024	-291	YJL101C	GSH1	Gsh1p
chrV	362398	362512	1560.900024	-645	YER102W	RPS8B	ribosomal 40S subunit protein S8B
chrVIII	450904	451015	1560.849976	194	YHR173C	YHR173C	hypothetical protein
chrXV	424438	424542	1557.900024	127	YOR050C		
chrXIII	224035	224154	1554.300049	-266	YML026C	RPS18B	ribosomal 40S subunit protein S18B

chrII	89622	89733	1553.099976	-121	YBL071C-B	YBL071C-B	hypothetical protein
chrVIII	462119	462233	1547.650024	-326	YHR179W	OYE2	Oye2p
chrII	392568	392681	1542.949951	-331	YBR077C	SLM4	Slm4p
chrV	222426	222534	1542.5	-159	YER035W	EDC2	Edc2p
chrXII	84210	84327	1535.75	-536	YLL028W	TPO1	Tpo1p
chrIV	117431	117534	1530.599976	-182	YDL191W	RPL35A	ribosomal 60S subunit protein L35A
chrIX	22172	22282	1528.800049	-3674	YIL172C	IMA3	Ima3p
chrXIV	763158	763262	1523	-618	YNR069C	BSC5	Bsc5p
chrIII	178331	178439	1519.449951	-165	YCR031C	RPS14A	ribosomal 40S subunit protein S14A
chrX	123110	123216	1518.600098	-215	YJL158C	CIS3	Cis3p
chrII	236086	236201	1518.600098	-349	YBL002W	HTB2	Htb2p
chrIV	130611	130753	1513.25	-198	YDL184C	RPL41A	ribosomal 60S subunit protein L41A
chrXV	901425	901527	1482.649902	-282	YOR312C	RPL20B	ribosomal 60S subunit protein L20B
chrII	407335	407438	1473.5	-217	YBR082C	UBC4	E2 ubiquitin-conjugating protein UBC4
chrXII	1028449	1028574	1472.050049	-343	YLR448W	RPL6B	ribosomal 60S subunit protein L6B
chrII	217192	217304	1467.349976	-119	YBL006C	LDB7	Ldb7p
chrXIV	503192	503298	1465.800049	-479	YNL065W	AQR1	Aqr1p
chrII	555857	555975	1464.099976	-633	YBR158W	AMN1	Amn1p
chrXVI	281806	281916	1462.199951	-261	YPL143W	RPL33A	ribosomal 60S subunit protein L33A
chrXII	282749	282850	1457.149902	-128	YLR075W	RPL10	ribosomal 60S subunit protein L10
chrV	284803	284951	1445.849976	-655	YER064C	VHR2	Vhr2p
chrIII	123589	123699	1445.400024	-641	YCR006C		

chrVIII	505877	505989	1438.25	-386	YHR204W	MNL1	Mnl1p
chrVIII	384814	384915	1433.649902	-646	YHR143W	DSE2	Dse2p
chrXIII	754426	754526	1431.550049	179	YMR242C	RPL20A	ribosomal 60S subunit protein L20A
chrIV	1278743	1278855	1429.699951	-411	YDR406W	PDR15	ATP-binding cassette multidrug transporter PDR15
chrXVI	425314	425465	1427.5	-293	YPL068C	YPL068C	hypothetical protein
chrIV	600048	600162	1425.600098	-688	YDR077W	SED1	Sed1p
chrXVI	502824	503000	1421.25	118	YPL025C		
chrXV	312689	312792	1416.849976	-373	YOL007C	CSI2	Csi2p
chrXI	231062	231179	1408.650024	-1107	YKL109W	HAP4	Hap4p
chrIX	127827	127946	1406.699951	-265	YIL123W	SIM1	Sim1p
chrXII	897920	898023	1393.75	-299	YLR387C	REH1	Reh1p
chrVII	384781	384917	1392.150024	-198	YGL063C-A		
chrXIII	751433	751534	1386.550049	-243	YMR240C	CUS1	Cus1p
chrXIII	675196	675318	1386.199951	-491	YMR205C	PFK2	6-phosphofructokinase subunit beta
chrXV	904003	904112	1374.300049	-401	YOR314W-A		
chrIV	1355690	1355800	1370.25	-192	YDR447C	RPS17B	ribosomal 40S subunit protein S17B
chrXI	326149	326290	1369.650024	448	YKL061W	BLI1	Bli1p
chrXVI	302905	303019	1368.25	-159	YPL131W	RPL5	ribosomal 60S subunit protein L5
chrXI	108988	109092	1362.650024	-229	YKL180W	RPL17A	ribosomal 60S subunit protein L17A
chrXVI	188724	188846	1362.5	-272	YPL189C-A	COA2	Coa2p
chrV	174817	174926	1358.800049	-377	YER011W	TIR1	Tir1p
chrIV	1401480	1401586	1358	-237	YDR471W	RPL27B	ribosomal 60S subunit protein L27B

chrXIII	653544	653670	1355.350098	-427	YMR195W	ICY1	Icy1p
chrV	153026	153167	1352.5	-424	YER001W	MNN1	Mnn1p
chrV	545185	545334	1348.849976	-352	YER177W	BMH1	Bmh1p
chrXV	1004227	1004345	1347.349976	-851	YOR355W	GDS1	Gds1p
chrXII	125194	125303	1343.649902	-286	YLL012W	YEH1	Yeh1p
chrXVI	67103	67213	1342.699951	-544	YPL256C	CLN2	Cln2p
chrXIII	510965	511080	1341.699951	53	YMR122C		
chrVII	856795	856928	1337.550049	-560	YGR180C	RNR4	ribonucleotide-diphosphate reductase subunit RNR4
chrIV	568935	569047	1333.399902	-288	YDR058C	TGL2	Tgl2p
chrXIV	662059	662194	1331.699951	-752	YNR016C	ACC1	acetyl-CoA carboxylase ACC1
chrII	604264	604410	1331.399902	-171	YBR189W	RPS9B	ribosomal 40S subunit protein S9B
chrII	60934	61043	1330.300049	-249	YBL087C	RPL23A	ribosomal 60S subunit protein L23A
chrII	466265	466410	1314.550049	-567	YBR112C	CYC8	Cyc8p
chrVIII	35959	36080	1295.150024	6	YHL033C	RPL8A	ribosomal 60S subunit protein L8A
chrVII	366078	366180	1292.449951	-133	YGL076C	RPL7A	ribosomal 60S subunit protein L7A
chrXII	65478	65595	1285.399902	-329	YLL039C	UBI4	ubiquitin
chrX	623064	623166	1282.199951	-101	YJR104C	SOD1	Sod1p
chrVII	650916	651020	1281.75	-351	YGR086C	PIL1	Pil1p
chrXI	533346	533477	1275.349976	53	YKR052C	MRS4	Mrs4p
chrX	159992	160104	1272.5	-201	YJL133C-A	YJL133C-A	hypothetical protein
chrVIII	293514	293634	1257.25	631	YHR095W		
chrV	322007	322114	1252.150024	-626	YER081W	SER3	phosphoglycerate dehydrogenase SER3
chrIV	1434497	1434597	1251.599976	-279	YDR490C	PKH1	Pkh1p

chrIV	369019	369137	1242.050049	-693	YDL047W	SIT4	Sit4p
chrXV	1028799	1028904	1232.300049	-226	YOR369C	RPS12	ribosomal 40S subunit protein S12
chrX	173127	173231	1230.699951	-812	YJL130C	URA2	Ura2p
chrXV	762189	762320	1224.400024	-170	YOR226C	ISU2	Isu2p
chrXII	665102	665237	1219.899902	-167	YLR259C	HSP60	chaperone ATPase HSP60
chrVII	766278	766386	1219.899902	606	YGR139W		
chrXIII	852188	852303	1216.949951	-385	YMR291W	TDA1	Tda1p
chrXV	384815	384916	1211.849976	265	YOR029W		
chrIV	721855	721970	1199.599976	-615	YDR133C		
chrIX	232664	232776	1198	-293	YIL068W-A		
chrXI	428546	428673	1192.449951	-58	YKL008C	LAC1	sphingosine N-acyltransferase LAC1
chrXIV	302459	302564	1192.449951	-169	YNL178W	RPS3	ribosomal 40S subunit protein S3
chrXI	463166	463280	1189.900024	-527	YKR011C	YKR011C	hypothetical protein
chrIV	1362422	1362534	1184.699951	-297	YDR451C	YHP1	Yhp1p
chrXII	39519	39635	1181.400024	-106	YLL051C	FRE6	Fre6p
chrXII	950693	950810	1160.050049	-278	YLR412C-A	YLR412C-A	hypothetical protein
chrX	69033	69168	1150.949951	-238	YJL194W	CDC6	AAA family ATPase CDC6
chrII	292037	292149	1150.050049	-228	YBR025C	OLA1	Ola1p
chrXI	431245	431385	1146.300049	-591	YKL006W	RPL14A	ribosomal 60S subunit protein L14A
chrVII	702960	703075	1142.449951	350	YGR107W		
chrV	335515	335681	1141.800049	-329	YER088W-B		
chrII	452252	452366	1134.650024	-340	YBR105C	VID24	glucose-induced degradation complex subunit VID24
chrX	525859	525976	1129.050049	-418	YJR048W	CYC1	Cyc1p
chrVII	987318	987437	1125.25	-672	YGR249W	MGA1	Mga1p

chrIV	556658	556799	1123.25	-256	YDR050C	TPI1	triose-phosphate isomerase TPI1
chrXVI	491689	491791	1122.900024	-376	YPL032C	SVL3	Svl3p
chrVII	271917	272031	1122.400024	-546	YGL125W	MET13	methylenetetrahydrofolate reductase (NAD(P)H) MET13
chrII	159876	159979	1120.800049	-234	YBL033C	RIB1	GTP cyclohydrolase II
chrVII	166154	166290	1109.099976	-1130	YGL178W	MPT5	Mpt5p
chrV	16082	16239	1109	-195	YEL071W	DLD3	Dld3p
chrXIII	347180	347300	1099.400024	-723	YMR037C	MSN2	Msn2p
chrII	614216	614316	1099.300049	-93	YBR196C-A	YBR196C-A	hypothetical protein
chrXIV	198220	198344	1097.25	-339	YNL241C	ZWF1	glucose-6-phosphate dehydrogenase
chrXIII	886612	886747	1096.300049	-107	YMR306C-A		
chrIV	1450918	1451035	1095.849976	-123	YDR500C	RPL37B	ribosomal 60S subunit protein L37B
chrII	681997	682097	1095.650024	1043	YBR231C	SWC5	Swc5p
chrXII	710591	710726	1087.599976	-522	YLR286C	CTS1	Cts1p
chrXI	614597	614727	1085.099976	-777	YKR092C	SRP40	Srp40p
chrIX	139279	139386	1082.550049	-420	YIL118W	RHO3	Rho3p
chrXVI	576184	576292	1078.900024	-314	YPR009W	SUT2	Sut2p
chrXVI	132373	132512	1075.449951	-327	YPL222C-A		
chrII	504543	504651	1073.800049	-257	YBR135W	CKS1	Cks1p
chrXIV	301795	301969	1073.75	-779	YNL179C		
chrXV	912014	912121	1072.099976	369	YOR318C		
chrIV	341325	341441	1063.099976	-236	YDL060W	TSR1	Tsr1p
chrXV	370984	371085	1054	-190	YOR020C	HSP10	Hsp10p
chrXIII	798076	798196	1048.75	-211	YMR265C	YMR265C	hypothetical protein
chrXV	520721	520840	1042.699951	-573	YOR107W	RGS2	Rgs2p
chrVII	1050308	1050489	1042.550049	-440	YGR279C	SCW4	Scw4p

chrV	123235	123349	1028.849976	-363	YEL017C-A	PMP2	Pmp2p
chrXIV	373780	373912	1024.349976	-265	YNL134C	YNL134C	hypothetical protein
chrVIII	21182	21339	1021.299988	-289	YHL040C	ARN1	Arn1p
chrXV	770214	770346	1020.050049	-520	YOR230W	WTM1	Wtm1p
chrIX	138333	138436	1018.699951	-507	YIL119C	RPI1	Rpi1p
chrX	195448	195572	1017.599976	-637	YJL116C	NCA3	Nca3p
chrXVI	404647	404764	1008.400024	-245	YPL081W	RPS9A	ribosomal 40S subunit protein S9A
chrXVI	770913	771021	1006.900024	-686	YPR119W	CLB2	Clb2p
chrXI	589676	589779	1005.299988	-422	YKR079C	TRZ1	Trz1p
chrXI	340303	340486	990.5	152	YKL052C	ASK1	Ask1p
chrIV	543739	543844	987.949951	-422	YDR043C	NRG1	Nrg1p
chrII	564951	565081	981.300049	-215	YBR162W-A	YSY6	Ysy6p
chrXIII	483588	483705	974.150024	-438	YMR108W	ILV2	acetolactate synthase catalytic subunit
chrXIII	353193	353302	972.599976	-624	YMR043W	MCM1	Mcm1p
chrXIII	119315	119431	972	-475	YML075C	HMG1	hydroxymethylglutaryl-CoA reductase (NADPH) HMG1
chrIV	1011529	1011702	966.950012	345	YDR274C		
chrIV	386910	387016	962.75	-1376	YDL037C	BSC1	Bsc1p
chrXII	789896	790027	957.800049	-715	YLR332W	MID2	Mid2p
chrXVI	280095	280222	954.099976	-322	YPL144W	POC4	Poc4p
chrVII	894412	894522	952.199951	-226	YGR198W	YPP1	Ypp1p
chrIV	307945	308070	943	-218	YDL083C	RPS16B	ribosomal 40S subunit protein S16B
chrIV	1429139	1429239	941.849976	-209	YDR487C	RIB3	3,4-dihydroxy-2-butanone-4-phosphate synthase RIB3
chrXIII	861206	861315	937.949951	-369	YMR296C	LCB1	serine C-palmitoyltransferase LCB1
chrX	348871	348999	937.849976	243	YJL047C-A	YJL047C-A	hypothetical protein

chrXIII	334967	335077	937.25	-276	YMR032W	HOF1	Hof1p
chrXII	674057	674169	930.200012	-169	YLR264C-A	YLR264C-A	hypothetical protein
chrII	798953	799073	930.150024	-491	YBR296C	PHO89	Pho89p
chrXV	40431	40532	929.699951	-267	YOL152W	FRE7	Fre7p
chrXIII	472975	473081	929.150024	236	YMR103C		
chrXVI	257130	257284	925.349976	-440	YPL156C	PRM4	Prm4p
chrV	342558	342662	921.050049	1	YER091C-A		
chrIV	909559	909677	915.299988	-436	YDR222W	YDR222W	hypothetical protein
chrXII	677793	677938	907.099976	-141	YLR266C	PDR8	Pdr8p
chrXII	962972	963077	903.049988	-761	YLR420W	URA4	dihydroorotase
chrXV	925249	925358	898.549988	-263	YOR324C	FRT1	Frt1p
chrVII	798239	798396	898.400024	-226	YGR155W	CYS4	cystathionine beta-synthase CYS4
chrXV	505408	505544	896.449951	-318	YOR096W	RPS7A	ribosomal 40S subunit protein S7A
chrIII	305739	305839	887.75	-322	YCR102C	YCR102C	hypothetical protein
chrXV	55235	55352	882.650024	-189	YOL143C	RIB4	lumazine synthase RIB4
chrVIII	422640	422780	880.699951	-362	YHR162W	MPC2	Mpc2p
chrXIV	427113	427255	880.549988	-430	YNL104C	LEU4	2-isopropylmalate synthase LEU4
chrV	431236	431336	878.5	-837	YER132C	PMD1	Pmd1p
chrXIII	872758	872867	868.849976	-187	YMR302C	YME2	Yme2p
chrVI	210605	210712	867.25	-279	YFR029W	PTR3	Ptr3p
chrXII	498646	498781	862.150024	-234	YLR167W	RPS31	ubiquitin-ribosomal 40S subunit protein S31 fusion protein
chrXIII	70213	70317	859.699951	127	YML100W-A	YML100W-A	hypothetical protein
chrXV	304909	305060	857.599976	-365	YOL011W	PLB3	Plb3p
chrXIII	500872	500979	857.049988	-237	YMR116C	ASC1	Asc1p
chrXVI	228035	228157	856	-218	YPL170W	DAP1	Dap1p

chrXV	825818	825919	853.049988	64	YOR268C	YOR268C	hypothetical protein
chrXIII	91151	91274	850.5	197	YML089C		
chrVIII	56055	56168	846.349976	-538	YHL024W	RIM4	Rim4p
chrXIV	652073	652181	841.799988	-338	YNR014W	YNR014W	hypothetical protein
chrXII	329437	329555	835.549988	-258	YLR094C	GIS3	Gis3p
chrVIII	133016	133134	833.549988	1028	YHR014W	SPO13	Spo13p
chrIX	420848	420950	832.549988	-163	YIR034C	LYS1	saccharopine dehydrogenase (NAD+, L-lysine-forming)
chrVIII	204331	204444	829.549988	-220	YHR048W	YHK8	Yhk8p
chrIV	930707	930822	826.450012	-365	YDR234W	LYS4	homoaconitate hydratase LYS4
chrXIII	608340	608452	825.849976	-185	YMR172C-A		
chrVIII	396939	397053	823.5	-255	YHR150W	PEX28	Pex28p
chrXII	818597	818710	822.75	-659	YLR344W	RPL26A	ribosomal 60S subunit protein L26A
chrVII	287365	287475	822.349976	1031	YGL118C		
chrX	36479	36595	818.799988	-288	YJL212C	OPT1	Opt1p
chrV	100118	100221	817.299988	-600	YEL027W	VMA3	Vma3p
chrV	242276	242386	808.549988	-830	YER045C	ACA1	Aca1p
chrVIII	512259	512369	801.300049	-418	YHR206W	SKN7	Skn7p
chrIX	84947	85104	800.950012	-28	YIL141W		
chrV	77702	77825	793.700012	-290	YEL040W	UTR2	Utr2p
chrIV	1345429	1345544	790.549988	-161	YDR442W		
chrXII	404252	404356	786.599976	-242	YLR130C	ZRT2	low-affinity Zn(2+) transporter ZRT2
chrXVI	689557	689659	786.549988	-499	YPR072W	NOT5	CCR4-NOT core subunit NOT5
chrXII	514958	515073	775.25	-247	YLR180W	SAM1	methionine adenosyltransferase SAM1

chrXI	490612	490756	770.650024	-680	YKR027W	BCH2	Bch2p
chrXIII	902554	902657	767.550049	-195	YMR315W	YMR315W	hypothetical protein
chrX	607973	608113	763.5	-262	YJR094W-A	RPL43B	Rpl43bp
chrXIV	350004	350108	757.950012	-300	YNL149C	PGA2	Pga2p
chrXV	891829	891941	756.799988	-453	YOR306C	MCH5	Mch5p
chrXII	263015	263122	756.400024	-126	YLR061W	RPL22A	ribosomal 60S subunit protein L22A
chrIX	177610	177748	746.25	2	YIL100C-A		
chrXVI	901680	901785	743.599976	-312	YPR184W	GDB1	bifunctional 4-alpha-glucanotransferase/amylo-alpha-1,6-glucosidase
chrIV	156087	156194	735	-178	YDL170W	UGA3	Uga3p
chrVII	677825	677971	734.450012	-797	YGR097W	ASK10	Ask10p
chrXIII	434405	434528	726.299988	-322	YMR083W	ADH3	alcohol dehydrogenase ADH3
chrXIII	170147	170278	721.550049	-190	YML052W	SUR7	Sur7p
chrXIII	37803	37919	721.550049	-335	YML116W	ATR1	Atr1p
chrII	326763	326869	717.049988	-757	YBR044C	TCM62	Tcm62p
chrXV	69173	69287	702.75	-476	YOL136C	PFK27	Pfk27p
chrIV	1256987	1257106	671.550049	-199	YDR390C	UBA2	E1 ubiquitin-activating protein UBA2
chrXV	832474	832645	668.450012	-254	YOR272W	YTM1	Ytm1p
chrXII	759161	759298	667.450012	-251	YLR312W-A	MRPL15	mitochondrial 54S ribosomal protein YmL15
chrXI	93849	93958	663.349976	-596	YKL185W	ASH1	Ash1p
chrVII	640560	640694	662.849976	-93	YGR079W	YGR079W	hypothetical protein
chrX	445081	445204	658.849976	-281	YJR004C	SAG1	Sag1p
chrXIII	747899	748001	657.200012	1597	YMR238W	DFG5	Dfg5p
chrXIII	667303	667407	640.450012	-182	YMR202W	ERG2	C-8 sterol isomerase ERG2
chrIX	370414	370527	631.150024	-234	YIR007W	YIR007W	hydrolase

chrXII	855615	855728	626.099976	-26	YLR364C-A		
chrIX	268002	268153	625	-232	YIL046W-A	YIL046W-A	hypothetical protein
chrII	101261	101378	616.349976	-163	YBL064C	PRX1	Prx1p
chrIV	562775	562956	614.349976	-538	YDR054C	CDC34	SCF E2 ubiquitin-protein ligase catalytic subunit CDC34
chrXVI	527104	527208	612.349976	-273	YPL015C	HST2	Hst2p
chrXVI	22600	22735	606.650024	-271	YPL274W	SAM3	Sam3p
chrII	697587	697702	604.550049	-342	YBR238C	YBR238C	hypothetical protein
chrVII	156307	156416	602.050049	-349	YGL184C	STR3	cystathionine beta-lyase STR3
chrVII	976969	977114	599.450012	-295	YGR243W	FMP43	Fmp43p
chrV	106956	107071	597.400024	-247	YEL024W	RIP1	ubiquinol--cytochrome-c reductase catalytic subunit RIP1
chrVIII	401139	401249	596.400024	-240	YHR152W	SPO12	Spo12p
chrVII	939166	939273	581.700012	-227	YGR221C	TOS2	Tos2p
chrI	198540	198708	579.550049	3163	YAR047C		
chrXIII	572879	572981	578.599976	401	YMR158C-A	YMR158C-A	hypothetical protein
chrII	662612	662714	566.300049	-414	YBR218C	PYC2	pyruvate carboxylase 2
chrII	628508	628660	559.599976	-584	YBR203W	COS111	Cos111p
chrXV	417976	418126	549.400024	-370	YOR047C	STD1	Std1p
chrX	268453	268577	518.800049	-284	YJL088W	ARG3	Arg3p
chrXIII	209141	209285	508	-312	YML034W	SRC1	Src1p
chrVII	206467	206572	472.200012	-514	YGL158W	RCK1	Rck1p
chrXVI	930516	930645	468.150024	-796	YPR196W	YPR196W	hypothetical protein
chrXVI	328957	329058	468.150024	-277	YPL117C	IDI1	isopentenyl-diphosphate delta-isomerase IDI1
chrIV	1056080	1056234	441.549988	-394	YDR297W	SUR2	sphingosine hydroxylase
chrIV	1087152	1087255	396.099976	-378	YDR312W	SSF2	Ssf2p
