# Supplementary material for: The replicative lifespan‐extending deletion of SGF73 results in altered ribosomal gene expression in yeast
Source: Aging Cell. 2017 May 31;16(4):785–96. doi: 10.1111/acel.12611 (PMC5506417; doi:10.1111/acel.12611)
Supplement: Supplementary file 7 — Table S2 Sgf73 and Ubp8 shared occupancy peaks from ChIP‐Seq analysis [file ACEL-16-785-s007.pdf]

Table S2. Sgf73 and Ubp8 shared occupancy peaks from ChIP-Seq analysis

Chr #	Start	End	Peak Score	Distance to TSS	Nearest PromoterID	Gene Name	Gene Description
chrI	141707	141838	3336.199951	-341	YAL005C	SSA1	Hsp70 family ATPase SSA1
chrXVI	645455	645583	3307.166748	-431	YPR036W-A	YPR036W-A	hypothetical protein
chrVII	772097	772212	2795.166748	-300	YGR142W	BTN2	Btn2p
chrXII	97654	97755	2784.233398	-219	YLL024C	SSA2	Hsp70 family chaperone SSA2
chrXIII	388438	388559	2773.300049	232	YMR057C		
chrXII	370573	370697	2680.233398	-156	YLR112W		
chrIV	1490147	1490262	2662.133301	-214	YDR524C-A		
chrVII	483464	483620	2627.06665	282	YGL007W		
chrVII	914977	915084	2583.033447	-211	YGR211W	ZPR1	Zpr1p
chrVII	371312	371427	2581.833252	-7	YGL072C		
chrXIV	619845	619948	2537.233398	-171	YNL006W	LST8	Lst8p
chrXI	518624	518748	2493.733398	-120	YKR040C		
chrXV	83246	83361	2493.300049	-383	YOL126C	MDH2	malate dehydrogenase MDH2
chrXV	619323	619443	2465.43335	-457	YOR153W	PDR5	ATP-binding cassette multidrug transporter PDR5
chrXII	368368	368532	2439.766602	-326	YLR108C	YLR108C	hypothetical protein
chrVII	609973	610076	2295.233398	-540	YGR060W	ERG25	methylsterol monooxygenase
chrVII	884045	884150	2236.199951	-287	YGR192C	TDH3	glyceraldehyde-3-phosphate dehydrogenase (phosphorylating) TDH3
chrIX	386713	386830	2228.833252	-1070	YIR018C-A	YIR018C-A	hypothetical protein
chrVI	221769	221875	2206.233398	-404	YFR031C-A	RPL2A	ribosomal 60S subunit protein L2A
chrXIII	306644	306751	2157.06665	-792	YMR017W	SPO20	Spo20p
chrIV	132841	132988	2148.600098	-523	YDL182W	LYS20	homocitrate synthase LYS20
chrVII	310724	310834	2139.766602	-188	YGL103W	RPL28	ribosomal 60S subunit protein L28
chrXV	877838	877959	2120.733398	-213	YOR298C-A	MBF1	Mbf1p
chrXVI	679114	679269	2108.56665	240	YPR064W		
chrXIV	739430	739581	2099.033203	-446	YNR060W	FRE4	Fre4p
chrXVI	785815	785935	2092.56665	-333	YPR124W	CTR1	Ctr1p
chrIV	1080572	1080682	2081.466797	-428	YDR309C	GIC2	Gic2p
chrXIII	632111	632218	2079.833252	-191	YMR186W	HSC82	Hsp90 family chaperone HSC82

chrIII	78427	78571	2046.366577	-580	YCL025C	AGP1	Agp1p
chrII	444793	444903	2012.166748	-155	YBR101C	FES1	Fes1p
chrXVI	40477	40599	2005.966675	-505	YPL265W	DIP5	Dip5p
chrV	306028	306150	1939.599976	-234	YER074W	RPS24A	ribosomal 40S subunit protein S24A
chrII	332532	332653	1938.93335	-239	YBR048W	RPS11B	ribosomal 40S subunit protein S11B
chrVII	726664	726769	1928.466675	-237	YGR117C	YGR117C	hypothetical protein
chrXI	99841	100000	1926.300049	-751	YKL182W	FAS1	tetrafunctional fatty acid synthase subunit FAS1
chrXV	109715	109895	1913.633301	-492	YOL109W	ZEO1	Zeo1p
chrII	415546	415649	1913.366699	-336	YBR084C-A	RPL19A	ribosomal 60S subunit protein L19A
chrXI	258357	258514	1902.5	478	YKL097C		
chrXIV	495272	495401	1884.766602	-335	YNL069C	RPL16B	ribosomal 60S subunit protein L16B
chrX	521717	521839	1868.633301	-176	YJR045C	SSC1	Hsp70 family ATPase SSC1
chrII	168066	168211	1836.93335	-285	YBL027W	RPL19B	ribosomal 60S subunit protein L19B
chrVII	149206	149324	1818.633301	251	YGL188C		
chrXII	568200	568315	1815.233276	-310	YLR214W	FRE1	Fre1p
chrXIII	302883	303024	1792.400024	-468	YMR015C	ERG5	C-22 sterol desaturase
chrV	225523	225632	1792.300049	-312	YER037W	PHM8	Phm8p
chrXIII	225563	225673	1786.833374	-253	YML025C	YML6	mitochondrial 54S ribosomal protein YmL6
chrXIII	915350	915483	1781.833374	-878	YMR319C	FET4	Fet4p
chrXIV	63156	63298	1781.366699	-284	YNL302C	RPS19B	ribosomal 40S subunit protein S19B
chrIII	122610	122748	1747.533325	324	YCR006C		
chrII	140560	140678	1737.633301	-359	YBL042C	FUI1	Fui1p
chrXIII	253438	253555	1735.833374	-224	YML007C-A	YML007C-A	hypothetical protein
chrXVI	98796	98904	1731.300049	-225	YPL240C	HSP82	Hsp90 family chaperone HSP82
chrIII	228859	228974	1730.233276	-394	YCR065W	HCM1	Hcm1p
chrXII	645883	646012	1722.5	6	YLR255C		
chrVII	23427	23542	1706.06665	-451	YGL253W	HXK2	hexokinase 2
chrIV	591702	591839	1686.866699	-426	YDR072C	IPT1	inositolphosphotransferase
chrXII	809351	809485	1683.333252	-579	YLR342W	FKS1	Fks1p
chrXII	241894	241998	1676.5	-286	YLR048W	RPS0B	ribosomal 40S subunit protein S0B

chrXIV	576314	576471	1674.699951	-335	YNL030W	HHF2	Hhf2p
chrXVI	794676	794786	1655.766724	-233	YPR131C	NAT3	Nat3p
chrIV	976552	976701	1646.133301	603	YDR260C	SWM1	Swm1p
chrXVI	653969	654090	1625.666626	-137	YPR043W	RPL43A	ribosomal 60S subunit protein L43A
chrIX	257361	257495	1614	-365	YIL052C	RPL34B	ribosomal 60S subunit protein L34B
chrV	396441	396542	1613.5	-278	YER117W	RPL23B	ribosomal 60S subunit protein L23B
chrXV	253915	254041	1608.733398	-319	YOL039W	RPP2A	ribosomal protein P2A
chrXIII	124357	124483	1604.333374	-248	YML073C	RPL6A	ribosomal 60S subunit protein L6A
chrX	75554	75706	1599.800049	-303	YJL189W	RPL39	Rpl39p
chrIV	321877	321984	1587.133301	-296	YDL075W	RPL31A	ribosomal 60S subunit protein L31A
chrXVI	453530	453639	1579.766602	151	YPL056C	LCL1	Lcl1p
chrV	141172	141289	1578.5	-412	YEL008C-A		
chrXV	987633	987812	1572.56665	1060	YOR348C	PUT4	Put4p
chrXIII	540470	540594	1567.300049	-476	YMR135C	GID8	glucose-induced degradation complex subunit GID8
chrXVI	432008	432191	1546.033325	204	YPL062W		
chrX	651426	651544	1542.599976	-416	YJR123W	RPS5	Rps5p
chrXIV	331036	331137	1538.900024	-236	YNL162W	RPL42A	ribosomal 60S subunit protein L42A
chrIV	357211	357333	1536.466675	-513	YDL055C	PSA1	mannose-1-phosphate guanylyltransferase
chrXII	88272	88433	1532.199951	-271	YLL026W	HSP104	chaperone ATPase HSP104
chrII	408680	408797	1527.099976	-431	YBR083W	TEC1	Tec1p
chrIV	1165017	1165208	1524.733276	-452	YDR345C	HXT3	Hxt3p
chrIV	892501	892622	1519.866699	-314	YDR214W	AHA1	Aha1p
chrVII	920127	920250	1516.533325	-387	YGR214W	RPS0A	ribosomal 40S subunit protein S0A
chrIV	974377	974492	1503.966675	-191	YDR258C	HSP78	chaperone ATPase HSP78
chrIV	491189	491321	1496.166626	238	YDR024W		
chrXIII	224035	224159	1481.833374	-269	YML026C	RPS18B	ribosomal 40S subunit protein S18B
chrXIII	661832	661966	1472.200073	-745	YMR199W	CLN1	Cln1p
chrIV	117426	117534	1471.699951	-184	YDL191W	RPL35A	ribosomal 60S subunit protein L35A
chrVII	516402	516521	1468.699951	-482	YGR014W	MSB2	Msb2p
chrXII	941022	941127	1467.300049	-409	YLR410W-A	YLR410W-A	gag protein

chrIV	550926	551096	1464.466675	-435	YDR046C	BAP3	Bap3p
chrXIII	26145	26277	1464.166626	207	YML122C		
chrXIV	444491	444598	1463.033325	-229	YNL096C	RPS7B	ribosomal 40S subunit protein S7B
chrXVI	281806	281916	1460.166626	-261	YPL143W	RPL33A	ribosomal 60S subunit protein L33A
chrV	362398	362512	1446.5	-645	YER102W	RPS8B	ribosomal 40S subunit protein S8B
chrIII	178331	178446	1444.400024	-168	YCR031C	RPS14A	ribosomal 40S subunit protein S14A
chrXII	737823	737998	1430.06665	-362	YLR304C	ACO1	aconitate hydratase ACO1
chrXII	1028449	1028574	1427	-343	YLR448W	RPL6B	ribosomal 60S subunit protein L6B
chrVII	398049	398174	1417.766724	-493	YGL056C	SDS23	Sds23p
chrIV	234405	234589	1415.233276	-430	YDL127W	PCL2	Pcl2p
chrXV	901416	901527	1414.199951	-277	YOR312C	RPL20B	ribosomal 60S subunit protein L20B
chrII	555857	555975	1410.333374	-633	YBR158W	AMN1	Amn1p
chrXII	282734	282850	1408.533325	-135	YLR075W	RPL10	ribosomal 60S subunit protein L10
chrXIII	880608	880722	1408	-433	YMR305C	SCW10	Scw10p
chrII	89610	89733	1404.400024	-115	YBL071C-B	YBL071C-B	hypothetical protein
chrXIII	754426	754546	1402.833374	189	YMR242W-A	YMR242W-A	hypothetical protein
chrIV	130611	130767	1397.699951	-205	YDL184C	RPL41A	ribosomal 60S subunit protein L41A
chrXVI	297165	297293	1390.133301	-324	YPL135W	ISU1	Isu1p
chrII	392561	392681	1386.266602	-328	YBR077C	SLM4	Slm4p
chrVIII	35959	36081	1354.466675	5	YHL033C	RPL8A	ribosomal 60S subunit protein L8A
chrXV	424438	424555	1333.800049	121	YOR050C		
chrXI	108988	109096	1314.333374	-227	YKL180W	RPL17A	ribosomal 60S subunit protein L17A
chrXV	904003	904186	1293.699951	-364	YOR314W-A		
chrVIII	384814	384924	1290.699951	-641	YHR143W	DSE2	Dse2p
chrIV	1401478	1401586	1282.833374	-238	YDR471W	RPL27B	ribosomal 60S subunit protein L27B
chrIX	127827	127946	1281.233398	-265	YIL123W	SIM1	Sim1p
chrXV	1004227	1004349	1267.233276	-849	YOR355W	GDS1	Gds1p
chrII	604264	604410	1265.099976	-171	YBR189W	RPS9B	ribosomal 40S subunit protein S9B
chrII	60934	61064	1264.366699	-260	YBL087C	RPL23A	ribosomal 60S subunit protein L23A
chrXIV	763150	763262	1262.266602	-614	YNR069C	BSC5	Bsc5p

chrVII	366078	366196	1259	-141	YGL076C	RPL7A	ribosomal 60S subunit protein L7A
chrX	236586	236717	1248.466675	-295	YJL101C	GSH1	Gsh1p
chrXVI	302885	303019	1244.266602	-169	YPL131W	RPL5	ribosomal 60S subunit protein L5
chrXIII	751418	751534	1236.599976	-236	YMR240C	CUS1	Cus1p
chrXVI	67098	67213	1232.899902	-541	YPL256C	CLN2	Cln2p
chrIV	600048	600199	1225.733398	-670	YDR077W	SED1	Sed1p
chrXIV	662057	662194	1205.699951	-751	YNR016C	ACC1	acetyl-CoA carboxylase ACC1
chrIV	1278743	1278884	1200.06665	-397	YDR406W	PDR15	ATP-binding cassette multidrug transporter PDR15
chrXI	533346	533477	1176.633301	53	YKR052C	MRS4	Mrs4p
chrXVI	425314	425472	1164.533325	-297	YPL068C	YPL068C	hypothetical protein
chrXV	1028782	1028904	1159.300049	-218	YOR369C	RPS12	ribosomal 40S subunit protein S12
chrIV	1434497	1434609	1149.466675	-285	YDR490C	PKH1	Pkh1p
chrV	153026	153167	1148.599976	-424	YER001W	MNN1	Mnn1p
chrIX	232664	232784	1142.099976	-289	YIL068W-A		
chrXIV	302459	302566	1110.033325	-168	YNL178W	RPS3	ribosomal 40S subunit protein S3
chrXII	65478	65605	1103.866699	-334	YLL039C	UBI4	ubiquitin
chrII	466265	466410	1100.599976	-567	YBR112C	CYC8	Cyc8p
chrXI	431245	431385	1056.56665	-591	YKL006W	RPL14A	ribosomal 60S subunit protein L14A
chrIV	341280	341441	1055	-259	YDL060W	TSR1	Tsr1p
chrIV	1362417	1362534	1045.266724	-294	YDR451C	YHP1	Yhp1p
chrXVI	404647	404764	1044.099976	-245	YPL081W	RPS9A	ribosomal 40S subunit protein S9A
chrXII	39519	39635	1043.06665	-106	YLL051C	FRE6	Fre6p
chrX	173123	173231	1033.333374	-810	YJL130C	URA2	Ura2p
chrV	335515	335693	1028	-323	YER088W-B		
chrXII	665102	665242	1023.299988	-170	YLR259C	HSP60	chaperone ATPase HSP60
chrXVI	770911	771021	1003.366699	-687	YPR119W	CLB2	Clb2p
chrXII	710591	710763	997.533325	-541	YLR286C	CTS1	Cts1p
chrII	504543	504652	994.033325	-257	YBR135W	CKS1	Cks1p
chrVIII	21175	21339	967.533325	-286	YHL040C	ARN1	Arn1p
chrVII	166154	166292	963.633362	-1129	YGL178W	MPT5	Mpt5p

chrIV	307945	308078	946.56665	-222	YDL083C	RPS16B	ribosomal 40S subunit protein S16B
chrXIII	886612	886747	929.56665	-107	YMR306C-A		
chrXII	498646	498781	875.633301	-234	YLR167W	RPS31	ubiquitin-ribosomal 40S subunit protein S31 fusion protein
chrXV	40416	40532	866.733337	-274	YOL152W	FRE7	Fre7p
chrXII	674056	674169	855.700012	-168	YLR264C-A	YLR264C-A	hypothetical protein
chrII	564951	565081	852.900024	-215	YBR162W-A	YSY6	Ysy6p
chrXV	925225	925358	840.299988	-251	YOR324C	FRT1	Frt1p
chrXIII	861206	861319	821.133301	-371	YMR296C	LCB1	serine C-palmitoyltransferase LCB1
chrXII	962972	963109	820.599976	-745	YLR420W	URA4	dihydroorotase
chrXII	263008	263122	819.333313	-129	YLR061W	RPL22A	ribosomal 60S subunit protein L22A
chrIV	1429139	1429254	815.233337	-216	YDR487C	RIB3	3,4-dihydroxy-2-butanone-4-phosphate synthase RIB3
chrXII	404252	404362	805.400024	-245	YLR130C	ZRT2	low-affinity Zn(2+) transporter ZRT2
chrXV	55228	55352	801.299988	-186	YOL143C	RIB4	lumazine synthase RIB4
chrXII	818597	818710	798.266663	-659	YLR344W	RPL26A	ribosomal 60S subunit protein L26A
chrVII	287365	287490	795.166687	1024	YGL118C		
chrV	100111	100221	754.899963	-603	YEL027W	VMA3	Vma3p
chrIX	84947	85104	746.833313	-28	YIL141W		
chrV	242276	242386	729.200012	-830	YER045C	ACA1	Aca1p
chrXI	490612	490773	692.56665	-672	YKR027W	BCH2	Bch2p
chrXII	855615	855770	678.533325	-47	YLR364C-A		
chrII	326763	326899	668.200012	-772	YBR044C	TCM62	Tcm62p
chrIV	1256977	1257106	640.700012	-194	YDR390C	UBA2	E1 ubiquitin-activating protein UBA2
chrXIII	572871	572981	541.833313	405	YMR158C-A	YMR158C-A	hypothetical protein

Ubp8 peaks not occupied by Sgf73

Chr #	Start	End	Peak Score	Distance to TSS	Nearest PromoterID	Gene Name	Gene Description
chrXV	779804	779904	1336.900024	-16	YOR235W		
chrVI	106322	106422	945.200012	-43	YFL015W-A		
chrIV	472272	472372	774.900024	469	YDR012W	RPL4B	ribosomal 60S subunit protein L4B
